# Loss of Non-motor Kinesin KIF26A Causes Congenital Brain Malformations via Dysregulated Neuronal Migration and Axonal Growth as well as Apoptosis

**DOI:** 10.1016/j.devcel.2022.09.011

**Published:** 2022-10-12

**Authors:** Xuyu Qian, Ellen M. DeGennaro, Maya Talukdar, Shyam K. Akula, Abbe Lai, Diane D. Shao, Dilenny Gonzalez, Jack H. Marciano, Richard S. Smith, Norma K. Hylton, Edward Yang, J. Fernando Bazan, Lee Barrett, Rebecca C. Yeh, R Sean Hill, Samantha Beck, Aoi Otani, Jolly Angad, Tadahiro Mitani, Jennifer E. Posey, Davut Pehlivan, Daniel Calame, Hatip Aydin, Osman Yesilbas, Kendall C. Parks, Emanuela Argilli, Eleina England, Kiho Im, Ajay Taranath, Hamish S. Scott, Christopher P. Barnett, Peer Arts, Elliott H. Sherr, James R. Lupski, Christopher A. Walsh

**Affiliations:** 1. Division of Genetics and Genomics, Boston Children's Hospital, Harvard Medical School, Boston, Massachusetts 02115, USA; 2. Howard Hughes Medical Institute, Boston Children's Hospital, Harvard Medical School, Boston, Massachusetts 02115, USA.; 3. Allen Discovery Center for Human Brain Evolution, Boston Children’s Hospital, Harvard Medical School, Boston, MA 02115, USA.; 4. Division of Health Sciences and Technology, Massachusetts Institute of Technology, Cambridge, MA 02142, USA.; 5. Harvard-MIT MD/PhD Program, Program in Neuroscience, Harvard Medical School, Boston, MA 02115, USA; 6. Department of Neurology, Boston Children's Hospital, Boston, MA 02115, USA.; 7. Department of Radiology, Boston Children’s Hospital, Boston, MA 02115, USA; 8. ℏ Bioconsulting LLC, Stillwater, MN 55082, USA; 9. Department of Neurobiology, Boston Children's Hospital, Harvard Medical School, Boston, MA 02115, USA; 10. Department of Molecular and Human Genetics, Baylor College of Medicine, Houston, Texas 77030, USA; 11. Department of Pediatrics, Baylor College of Medicine, Houston, Texas 77030, USA; 12. Texas Children's Hospital, Houston, Texas 77030, USA; 13. Section of Pediatric Neurology and Developmental Neuroscience, Department of Pediatrics, Baylor College of Medicine, Houston, TX 77030, USA; 14. Centre of Genetics Diagnosis, Zeynep Kamil Maternity and Children's Training and Research Hospital, Istanbul, Turkey; Private Reyap Istanbul Hospital, Istanbul -, Turkey.; 15. Department of Pediatrics, Division of Pediatric Critical Care Medicine, Faculty of Medicine, Karadeniz Technical University, Trabzon 61080, Turkey; 16. Department of Neurology, University of California, San Francisco, San Francisco, CA 94143, USA; 17. Center for Mendelian Genomics, Program in Medical and Population Genetics, Broad Institute of MIT and Harvard, Cambridge, Massachusetts 02142, USA; 18. Division of Newborn Medicine, Boston Children's Hospital, Harvard Medical School, Boston, Massachusetts 02115, USA; 19. Department of Medical imaging, South Australia Medical Imaging, Women's and Children's Hospital, North Adelaide, SA, Australia; 20. Department of Genetics and Molecular Pathology, Centre for Cancer Biology, An Alliance Between SA Pathology and the University of South Australia, Adelaide, Australia.; 21. Adelaide Medical School, University of Adelaide, Adelaide, SA, Australia; 22. ACRF Cancer Genomics Facility, Centre for Cancer Biology, An Alliance Between SA Pathology and the University of South Australia, Adelaide, SA, Australia.; 23. Australian genomics, Parkville, Victoria, Australia; 24. Pediatric and Reproductive Genetics Unit, Women's and Children's Hospital, North Adelaide, SA, Australia; 25. Institute of Human Genetics and Weill Institute for Neurosciences, University of California, San Francisco, San Francisco, CA 94143, USA; 26. Human Genome Sequencing Center, Baylor College of Medicine, Houston, Texas 77030, USA; 27. These authors contributed equally; 28. Lead contact christopher.walsh@childrens.harvard.edu

## Abstract

Kinesins are canonical molecular motors but can also function as modulators of intracellular signaling. KIF26A, an unconventional kinesin that lacks motor activity, inhibits growth factor receptor bound protein 2 (GRB2)- and focal adhesion kinase (FAK)-dependent signal transduction, but its functions in the brain have not been characterized. We report a patient cohort with biallelic loss-of-function variants in *KIF26A*, exhibiting a spectrum of congenital brain malformations. In the developing brain, *KIF26A* is preferentially expressed during early and mid-gestation in excitatory neurons. Combining mice and human iPSC-derived organoid models, we discovered that loss of *KIF26A* causes excitatory neuron-specific defects in radial migration, localization, dendritic and axonal growth, and apoptosis, offering a convincing explanation of the disease etiology in patients. Single-cell RNA-sequencing in *KIF26A* knock-out organoids revealed transcriptional changes in MAPK, MYC and E2F pathways. Our findings illustrate the pathogenesis of *KIF26A* loss-of-function variants, and identify the surprising versatility of this non-motor kinesin.

## INTRODUCTION

The kinesin superfamily proteins are canonical molecular motors that transport protein complexes or organelles along microtubules by ATP hydrolysis (Niwa, 2015). Through their interactions with microtubules and regulation of microtubule dynamics, many KIFs are involved in cellular processes such as mitosis, migration and neurite outgrowth (Niwa, 2015). In addition, some kinesins play additional roles in signal transduction by regulating the intracellular localization of signal protein complexes and mediating the strength of the signal (Konjikusic et al., 2021; Midorikawa et al., 2006). Abnormal kinesin functions have been associated with a variety of neurodevelopmental diseases (Asselin et al., 2020; Geng et al., 2018; Hirokawa et al., 2010; Janisch et al., 2013; Mukherjee et al., 2020; Zhou et al., 2013).

*KIF26A* encodes a member of the Kinesin-11 family, which also includes *KIF26B*, *SMY1* and *VAB8* (Lawrence et al., 2004; Miki et al., 2005). However, KIF26A does not function in microtubule-based transport, since its divergent motor domain can bind to microtubules but cannot hydrolyze ATP (Zhou et al., 2009). In mouse enteric neurons, Kif26a directly binds to the signal transduction adaptor protein Grb2 and inhibits Grb2/SHC complex formation, thereby disrupting RET tyrosine kinase activation and subsequent signaling through the Ras-MAPK and PI3K-Akt cascades (Besset et al., 2000; Zhou et al., 2009). Kif26a also recruits focal adhesion kinase (FAK) to cytoplasmic microtubules, sequestering FAK from phosphorylation by integrin-activated Src family kinase (SFK) (Calalb et al., 1995; Wang et al., 2018). In addition, via its binding to microtubules, Kif26a functions as a microtubule stabilizer such that loss of Kif26a causes dysregulation of microtubule dynamics (Zhou et al., 2009). *Kif26a* knockout (KO) mice exhibit megacolon, enteric neuronal hyperplasia and defective neurite outgrowth (Zhou et al., 2009), but the role of *KIF26A* in the central nervous system has not yet been characterized. Here, we show that biallelic variants in human *KIF26A* are associated with a spectrum of congenital brain malformations. Combining *in vivo* animal and *in vitro* human brain organoid models, we found that KIF26A is critical for normal brain development, not as a microtubule-based motor, but instead as a modulator of intracellular signaling pathways that control radial migration, neurite and axon growth, and apoptosis of cortical excitatory neurons.

## RESULTS

### Identification of Biallelic Variants in *KIF26A* in Patients with Congenital Brain Malformations

**Figure 1.**
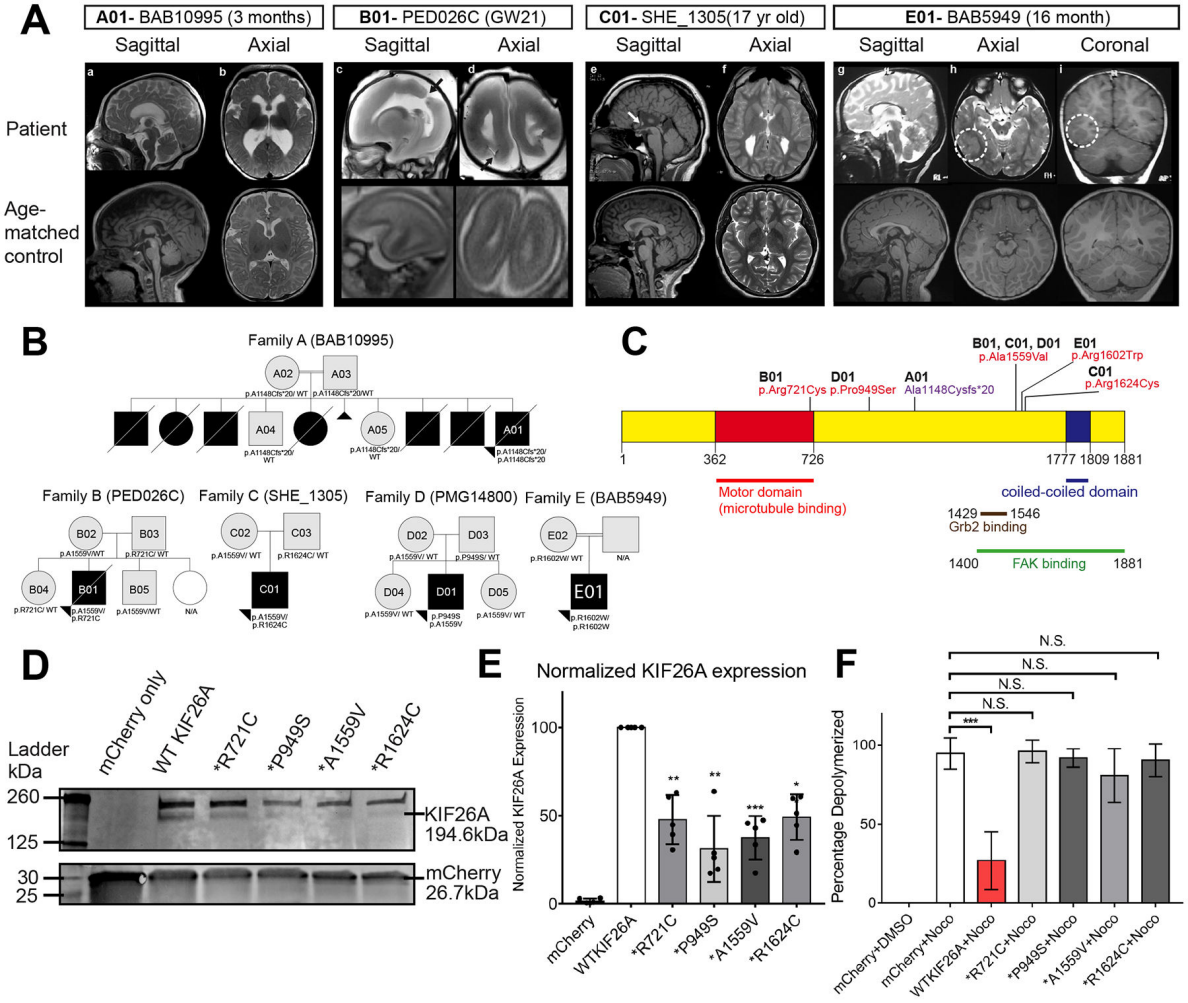
Biallelic Variants in *KIF26A* Associated with Congenital Brain Malformations Disrupt Protein Expression and Function. **(A)** MRI images showing brain abnormalities in human subjects with biallelic *KIF26A* variants (top) and plane-matched images from age-matched control brains(bottom). Sagittal (a) and axial (b) T2-weighted images of subject A01 at 3 months of age demonstrate reduced cerebral white matter volume (notably the corpus callosum (CC)) with supratentorial ventriculomegaly and cerebral atrophy. Sagittal (c) and axial (d) T2 weighted postmortem brain MRI of B01 (pregnancy ended at gestational week (GW) 21) demonstrate full thickness defects affecting the parietal lobes (black arrows). Note that tissue decomposition/swelling distorts brain morphology. Sagittal T1 (e) and axial T2 (f) images of subject C01 at 17 years of age demonstrate complete agenesis of the CC with preservation of the anterior commissure (white arrow) and colpocephaly. Mid-sagittal T2 weighted (g), axial T2 weighted (h), and coronal T1 weighted images (i) of subject E01 demonstrate increased thickness, gyral frequency, and haziness of the inferior right temporal cortex (circled in h and i) consistent with cortical dysplasia and polymicrogyria, with thinning of the CC (g). **(B)** Family pedigrees of affected individuals. Square, male; circle, female; black shading, affected (or presumably affected, not genotyped) individual. Those individuals who were genotyped are labeled. Double horizontal lines indicate consanguineous parents. “NA” indicates genotype information was not available. See also, Table S1. **(C)** Protein domains of KIF26A. Variants from affected individuals are annotated on the corresponding amino acid positions. **(D)** Western blot of transfected HEK293T lysate showing expression of KIF26A and mCherry tag upon transfection of WT and patient variant KIF26A expression plasmids. Note that the lower molecular weight band of KIF26A is of the correct molecular size of KIF26A protein (194.6kDa) and is therefore considered the authentic band used in quantification. **(E)** Quantification of relative expression levels of WT and variant KIF26A from Western blots on HEK293T cells transfected with expression plasmids. mCherry expressed from the same plasmids is used to normalize the differences in transfection efficiency. Dots represent independent biological replicates, bars represent Mean ± S.D. (n = 5 independent experiments. Student’s t-test: **, p < 0.005, ***, p < 0.0005). **(F)** Microtubule depolymerization assay on SHSY5H cells transfected with WT and variant KIF26A. Shown is the quantification for the percentage of transfected cells exhibiting Nocodazole (Noco)-induced microtubule depolymerization acutely after 15 minutes of 10μM Noco treatment. Values represent Mean ± S.D. (n = 10 areas of view for each condition. Student’s t-test: ***, p < 0.0005, N.S., not significant). See also Figures S1, S2 and Table S1.

As part of a rare variant, family-based genomics approach by exome sequencing (ES) to identify genes associated with brain malformations, we found 5 unrelated subjects with congenital brain malformations who had inherited biallelic mutations in *KIF26A* (NM_015656.1, hg19) (Figure 1A and B) but no other rare, segregating, or likely damaging candidate variant alleles (Table S1). A01, the proband of Family A (BAB10995), who was recently identified among a Turkish neurodevelopmental disorder cohort (Mitani et al., 2021), presented with microcephaly (−3.45SD) and an MRI suggesting a component of cerebral atrophy, as well as dysmorphic features and ileus with megacolon. Brain MRI at 3 months of age showed reduced white matter, ventriculomegaly and a thin corpus callosum (CC) (Figure 1A). This consanguineous family had six other children who died in infancy for whom limited clinical records indicated that several of them shared central nervous system (CNS) and gastrointestinal abnormalities (Figure 1B). ES revealed an inherited homozygous frameshift variant in *KIF26A* c.3440dupC, p.Ala1148Cysfs*20 (Figure S1A), predicted to cause complete loss of KIF26A expression. This variant is absent in normal individuals in the gnomAD v3 database. B01 from Family B (PED026C), was diagnosed prenatally with bilateral schizencephaly at 21 weeks gestation. Other abnormalities included shortened CC (−3.1 SD), high arched palate and long philtrum, and absence of a stomach bubble on ultrasound (Table S1). MRI from fetopsy after termination confirmed the bilateral schizencephaly and further revealed abnormal grey-white matter differentiation, with an abnormally fused molecular layer (Figure 1A). ES revealed biallelic, compound heterozygous variants in *KIF26A*: c.2161C>T, p.Arg721Cys, and c.4676C>T, p.Ala1559Val, segregating in accordance with Mendelian expectations for an autosomal recessive (AR) disease trait (Figure S1B). C01 from Family C (SHE_1305), is a male born to non-consanguineous parents with mild developmental delay and learning disability. A brain MRI at 18 years demonstrated agenesis of the CC and an enlarged anterior commissure, absent hippocampal commissure, colpocephaly, decreased white matter volume, and present Probst Bundles (Figure 1A). ES revealed inherited compound heterozygous variants in *KIF26A*: c.4676C>T, p.Arg1624Cys, and c.4870C>T, p.Ala1559Val (Figure S1C). D01 from Family D (PMG_14800) was diagnosed with polymicrogyria and hydrocephalus, with inherited compound heterozygous variants in *KIF26A*: c.2845C>T, p.Pro949Ser, and c.4676C>T, p.Ala1559Val (Figure S1D). MRI images from D01 were not available for review. E01 from Family E (BAB5949) is a male born to consanguineous parents with growth retardation and developmental delay. Brain MRI performed at 18 months revealed a thin CC, ventriculomegaly and polymicrogyria (Figure 1A). Exome sequencing demonstrated homozygous variants in *KIF26A*: c.4804C>T; p.Arg1602Trp (Figure S1E).

The missense variants identified are distributed widely in the protein but all involve amino acids that are highly conserved across mammalian species (Figure S1F), suggesting - along with the mode of inheritance - a loss-of-function (LoF) model of disease. Gain-of-function or “dominant negative” is highly unlikely, because none of the parents or siblings carrying heterozygous variants exhibited any neurological abnormalities (Figure 1B and Figure S1A-E). There are three discernable globular domains in KIF26A predicted by AlphaFold 2 (Jumper et al., 2021), but most of the identified variants reside in the disordered chain between the kinesin motor domain and a C-terminal helical module implicated in FAK binding (Figure S1G and H). The c.4676C>T, p.Ala1559Val variant shared by 3 of our subjects is present in normal individuals in the gnomAD v3 database (AF: 0.000975) but no homozygotes are observed, consistent with a heterozygous carrier state for a recessive disorder. Overall, these five families associate biallelic *KIF26A* mutations with corpus callosum defects, ventricular abnormalities, cortical malformations on the polymicrogyria/schizencephaly spectrum and additional variable brain and systemic abnormalities.

### Patient *KIF26A* missense variants express at reduced level and lose microtubule stabilizing function

The diverse biallelic mutations, observed AR inheritance pattern, and absence of neurological phenotypes in carrier parents in the cohort lead to our hypothesis that the pathogenesis of *KIF26A* mutations is due to LoF. We transfected expression constructs for wild-type (WT) and patient variant forms of *KIF26A* in HEK293T cells, a cell type with no baseline *KIF26A* expression, and found that WT and all 4 tested variants can be expressed exogenously from the plasmid, and the variant proteins displayed normal cytoplasmic localization (Figure S2A). However, Western blot revealed that all of the variant KIF26As expressed at significantly lower levels than WT KIF26A (Figure 1D and E), supporting our hypothesis that the patient variants cause partial LoF likely due to destabilization of the protein.

In human neuroblastoma cell line SHSY5Y, overexpression of WT KIF26A, but not variant KIF26As, stabilizes microtubules, making them resistant to depolymerization induced by Nocodazole treatment, a small molecule commonly used for this purpose (Figure 1F and Figure S2B) (Vasquez et al., 1997). This loss of the microtubule stabilizing function of KIF26A further supports the pathogenicity of the missense variants. As SHSY5Y cells have high baseline KIF26A expression, we also tested the effect of partial KIF26A loss by shRNA-induced knock-down (KD) and found that KIF26A depletion reduced the abundance of acetylated tubulin and detyrosinated tubulin, indicating that normal KIF26A expression is required to maintain microtubule stability in these neuron-like cells (Figure S2D and E). Missense proteins produced from the variants often have altered configuration and stability, which may underlie the reduced expression level and loss of microtubule stabilizing function in the KIF26A variants. We evaluated the impacts of the variants using a machine learning-based computational predictor, MUpro(Cheng et al., 2006) and the prediction results showed with high confidence values that the patient KIF26A missense variants would likely decrease protein stability and predict deleterious effects (Figure S2C). Together, these results provide strong evidence that the missense variants discovered in our patients impair KIF26A function, consistent with the AR inheritance pattern from our genetic analysis.

### *KIF26A* is Preferentially Expressed by Migrating Excitatory Neurons in the Developing Cerebral Cortex

**Figure 2.**
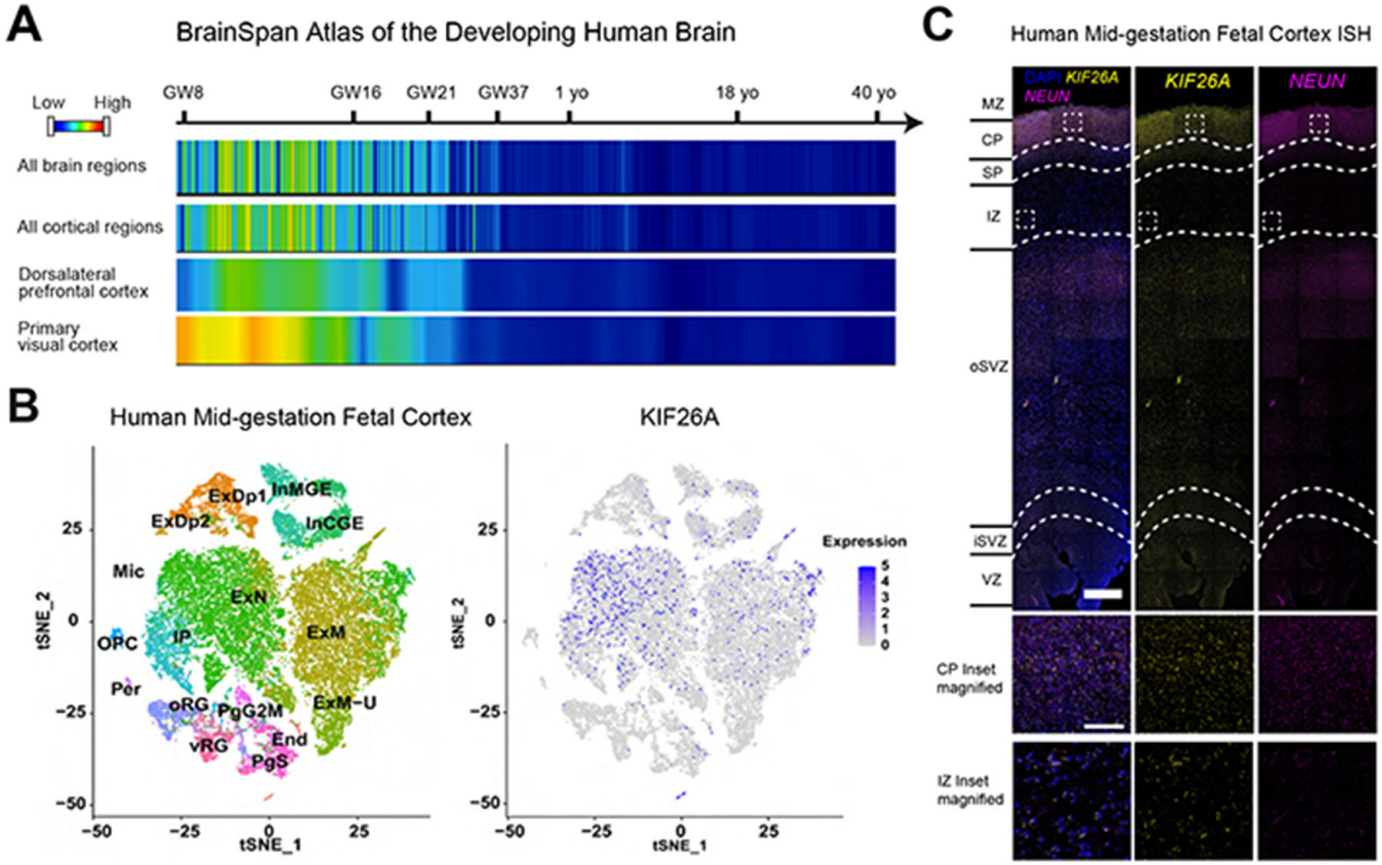
*KIF26A* is Preferentially Expressed by Migrating Excitatory Neurons in the Developing Cerebral Cortex. **(A)** Relative expression of *KIF26A* throughout human embryonic and postnatal brain development. Development progresses temporally from left to right, with key developmental times annotated on the axis. GW, gestational week; yo, year old (after birth). Data obtained from BrainSpan Atlas of the Developing Human Brain (Miller et al., 2014). **(B)** Cell-type clusters (left) and feature plot showing *KIF26A* expression (right) in human GW17-18 fetal cortex single-cell RNA-seq. Dataset and cluster annotations obtained from Polioudakis et al., 2019. The clustering and annotation from the original publication are kept unchanged. End, endothelial cells; PgS, progenitors in S phase; PgG2M, progenitors in G2M phase; vRG, ventricular radial glia; oRG, outer radial glia; Per, pericytes; OPC, oligodendrocyte precursor cells; IP, intermediate progenitor; ExN, migrating excitatory neurons; ExM, maturing excitatory neurons; ExM-U, maturing upper layer excitatory neurons; ExDp, deep layer excitatory neurons; InMGE, medial ganglionic eminence interneurons; InCGE, caudal ganglionic eminence interneurons. **(C)** *In situ* hybridization for *KIF26A* and *RBFOX3*(NeuN) on GW22 medial cortex. Bottom shows magnified view of the CP and the IZ indicated by the squares. Hybridization the CP and IZ is consistent with scRNAseq data suggesting expression in migrating and maturing excitatory neurons. Scale bars = 500μm (top), = 100 μm (bottom). MZ, marginal zone; CP, cortical plate; SP, subplate; IZ, intermediate zone; oSVZ, outer subventricular zone; iSVZ, inner subventricular zone; VZ, ventricular zone.

Analysis of the developmental transcriptome data from the BrainSpan Atlas of the Developing Human Brain (Miller et al., 2014) showed that *KIF26A* expression was enriched in the first and second trimesters (up to gestational week (GW) 26) of embryonic development, with expression decreasing rapidly entering the third trimester and becoming undetectable after birth (Figure 2A). This expression pattern was consistent across the anterior-posterior axis in the cortex, and temporally overlaps with the primary periods of the radial migration of cortical excitatory neurons (Rakic, 2009). Analysis of single nucleus RNA sequencing (snRNA-seq) datasets of mid-gestation (GW17-18) human fetal cortex (Polioudakis et al., 2019) revealed that *KIF26A* is predominantly expressed in excitatory neurons, with low expression in at least a fraction of intermediate progenitor cells (IPCs) and little expression in inhibitory neurons and radial glias (Figure 2B). Interestingly, *KIF26A* expression was more enriched in the Migrating Excitatory Neurons (ExN) cluster compared to the Maturing Excitatory Neurons (ExM) cluster (Figure 2B), suggesting a reduction in *KIF26A* expression as cortical neurons mature after completing migration, consistent with the temporal dynamics in bulk samples. We validated the expression of *KIF26A* in the cortical plate (CP), subplate (SP) and intermediate zone (IZ) of mid-gestation human fetal cortex using *in situ* hybridization (ISH) (Figure 2C). Kif26a expression was detected with a similar pattern in E16.5 mouse cortex (Figure S3A), suggesting potentially conserved roles of *KIF26A* in rodent and human brain development.

### *Kif26a* loss Disrupts Radial Migration of Cortical Excitatory Neurons

**Figure 3.**
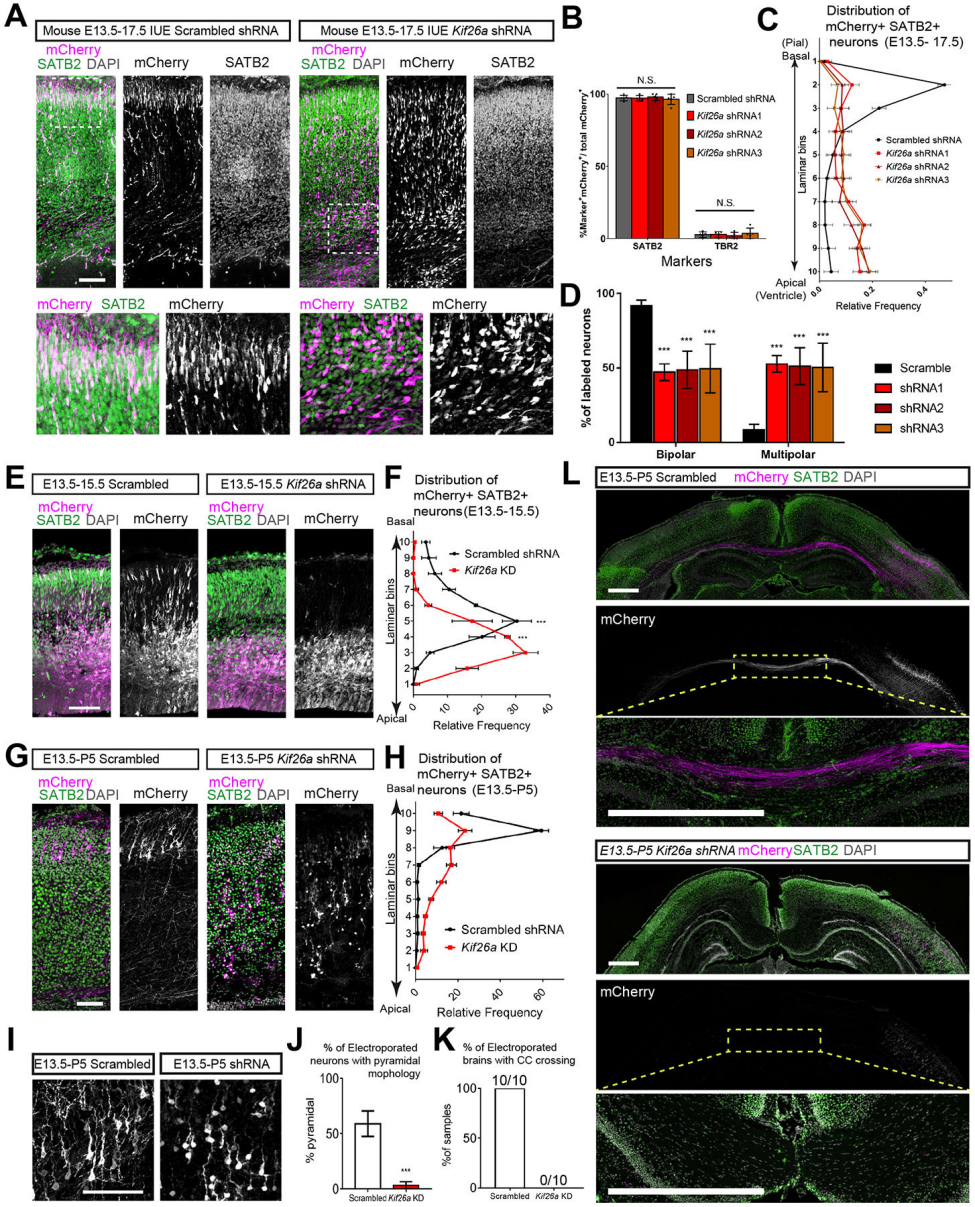
Kif26a Regulates Neuronal Radial Migration, Terminal Localization, Morphology and Corpus Callosum Development. **(A)** Representative images of *in utero* electroporation (IUE) of control scrambled shRNA (left) and *Kif26a* shRNA (right) in mouse cortex at E13.5 and analyzed at E17.5 (E13.5-17.5, 4 days post electroporation). Bottom magnified view show electroporated cells are SATB2^+^ neurons. Scale bars = 100μm. **(B)** Quantification of the percentage of mCherry-labeled cells expressing neuronal marker SATB2 or IPC marker TBR2. Values represent Mean ± S.D. (n = 5 brains for scrambled, n = 4 for shRNA1, n=6 for shRNA2, n = 5 for shRNA3. Student’s t-test: N.S., not significant). **(C)** Quantification of the laminar distributions of electroporated neurons in the cortex for E13.5-17.5. The cortex was evenly divided into 10 bins from basal (bin 1) to apical (bin 10) surfaces and the cell distribution was normalized by the total number of electroporated cells in the analyzed area. Only mCherry^+^, SATB2^+^ neurons are quantified. Values represent Mean ± S.D. (n = 7 brains for scrambled, n = 6 for shRNA1 and shRNA2, n = 5 for shRNA3). **(D)** Quantification of the percentage of labeled neurons displaying bipolar or multipolar morphology, showing loss of bipolar morphology with *Kif26a* deficient neurons. Values represent Mean ± S.D. (Same samples as **(C)**. Student’s t-test: **, p < 0.005; ***, p < 0.0005). **(E)** Representative images of IUE in mouse cortex at E13.5 and analyzed at E15.5 (E13.5-15.5, 2 days post electroporation). Scale bar = 100μm. **(F)** Quantification of the laminar distributions of electroporated neurons in the cortex for E13.5-15.5. Similar to **(C).** Only mCherry+, SATB2+ neurons are quantified. Values represent Mean ± S.D. (n = 7 brains for scrambled, n = 4 for shRNA KD. Student’s t-test: ***, p < 0.0005). **(G)** Representative images of IUE in mouse cortex at E13.5 and analyzed at P5 (E13.5-P5, 10 days post electroporation). Scale bar = 100μm. **(H)** Quantification of the laminar distributions of electroporated neurons in the cortex for E13.5-P5. Similar to **(C).** Only mCherry+, SATB2+ neurons are quantified. Values represent Mean ± S.D. (n = 10 brains). **(I)** Representative magnified images showing morphology of electroporated neurons at P5. Scale bar = 100μm. **(J)** Quantification of the percentage of electroporated neurons exhibiting pyramidal morphology at P5. Pyramidal morphology is defined as neurons having 1 basal dendrite and at least 2 apical dendrites from the soma. Values represent Mean ± S.D. (n = 10 brains. Student’s t-test: ***, p < 0.0005). **(K)** Quantification of the percentage of P5 brains with electroporated axons crossing the corpus callosum (CC). All (10 out of 10) scrambled shRNA brains showed CC crossing, while none (0 out of 10) of *Kif26a* KD brains showed CC crossing. **(L)** Representative images showing electroporated neurons send axons across the corpus callosum to the contralateral hemisphere only in scrambled control (top), but not in *Kif26a* KD (bottom). Insets show magnified view of selected areas. Scale bar = 500μm. See also Figures S3 and S4.

We depleted Kif26A from migrating cortical neurons in mice using *in utero* electroporation (IUE) of E13.5 mice with three short hairpin RNAs (shRNA) validated by RT-PCR (Figure S3B), and observed defective excitatory neuronal migration. Four days after IUE (E17.5) into the dorsal forebrain, the vast majority (~98%) of mCherry^+^ electroporated cells expressed the late-born cortical neuron marker Satb2, while a very small proportion expressed IPC marker Tbr2 (~2%) (Figure S3C). This proportionality is consistent across control and *Kif26a* KD, indicating that *Kif26a* KD did not impede neuronal differentiation (Figure 3B). *Kif26a* KD induced by all three shRNAs caused strong radial migration defects compared to scrambled shRNA controls, with many KD neurons stalled in the subventricular zone (SVZ) and the ventricular zone (VZ), while the control neurons reached the upper layer of the cortical plate (CP) (Figure 3A and C). Notably, this defect was related to radial migration but not neuronal differentiation or fate choice, because despite their abnormal localization, the KD cells stalled in the SVZ and VZ expressed Satb2 but not Tbr2 (Figure S3C), and many KD neurons exhibited multipolar morphology instead of normal bipolar morphology, a deficit characteristic of impaired radial migration (Figure 3A and D) (Noctor et al., 2004). While not as pronounced as E17.5, the migratory defect was also detected earlier at E15.5 (2 days after IUE), as quantitative analysis showed abnormal laminar localization of KD neurons (Figure 3E and F). Similarly, overexpression (OE) of human KIF26A in the mouse cortex also caused a strong migrational defects with many OE neurons arrested in the VZ or SVZ, suggesting that abnormally increased Kif26a expression may also be detrimental (Figure S3D and E). Lastly, we examined cell death in control and *Kif26a*-KD brains using TUNEL staining and found no significant differences at either E15.5 or E17.5 (Figure S4A-D). Our embryonic mouse data provides strong evidence that Kif26a is essential for radial migration of cortical excitatory neurons specifically, and that loss of Kif26a disrupts migration in a cell-autonomous manner.

### *Kif26a* Loss Impairs Neuronal Localization, Morphology and CC Development in Mouse Brain

Examination of electroporated mouse brains at P5 (10 days after IUE) revealed that the defect of migration is permanent, leading to a lasting mis-localization of excitatory neurons. While control electroporated neurons localized to the upper layers (II and III) of the cortex and formed a condensed uniform layer, *Kif26a* KD neurons failed to reach the same destination and were scattered throughout layers II to VI (Figure 3G and H), revealing that the migratory defect persisted to result in impaired terminal localization postnatally. Moreover, control electroporated neurons showed developed pyramidal morphology with a basal dendrite and multiple apical dendrites, as well as visible long descending axons. In striking contrast, KD neurons mostly retained an immature morphology with short basal dendrites (Figure 3I and J). Since the neurons born around E13.5 were mostly upper layer callosal projection neurons, control mCherry^+^ neurons at P5 sent out long axons that formed the axonal bundle to cross the CC and reach the contralateral hemisphere. However, CC crossing was completely blocked in *Kif26a* KD neurons with no mCherry-labeled axons found in the axonal bundle (Figure 3K and L). This observation was fully consistent across all the samples we examined at P5, as 100% (10 out of 10 mice) of control IUE brains and 0% (0 out of 10 mice) of *Kif26a* KD brains showed mCherry-labeled axons crossing the CC (Figure 3K). Nevertheless, the overall morphology of the CC appeared normal in the brains electroporated with *Kif26a* shRNA (Figure 3L), demonstrating that Kif26a loss disrupted axonal development cell-autonomously in electroporated neurons only, as the majority of cells were not electroporated and thus the gross CC formation was unaffected. These results illustrated that normal Kif26a function is required for CC development and this dependence establishes a strong association between *KIF26A* LoF and the CC abnormalities seen in our patients (Table S1).

### *KIF26A* Knockout Disrupts Neurite Outgrowth and Motility in Human iPSC-derived Neurons

**Figure 4.**
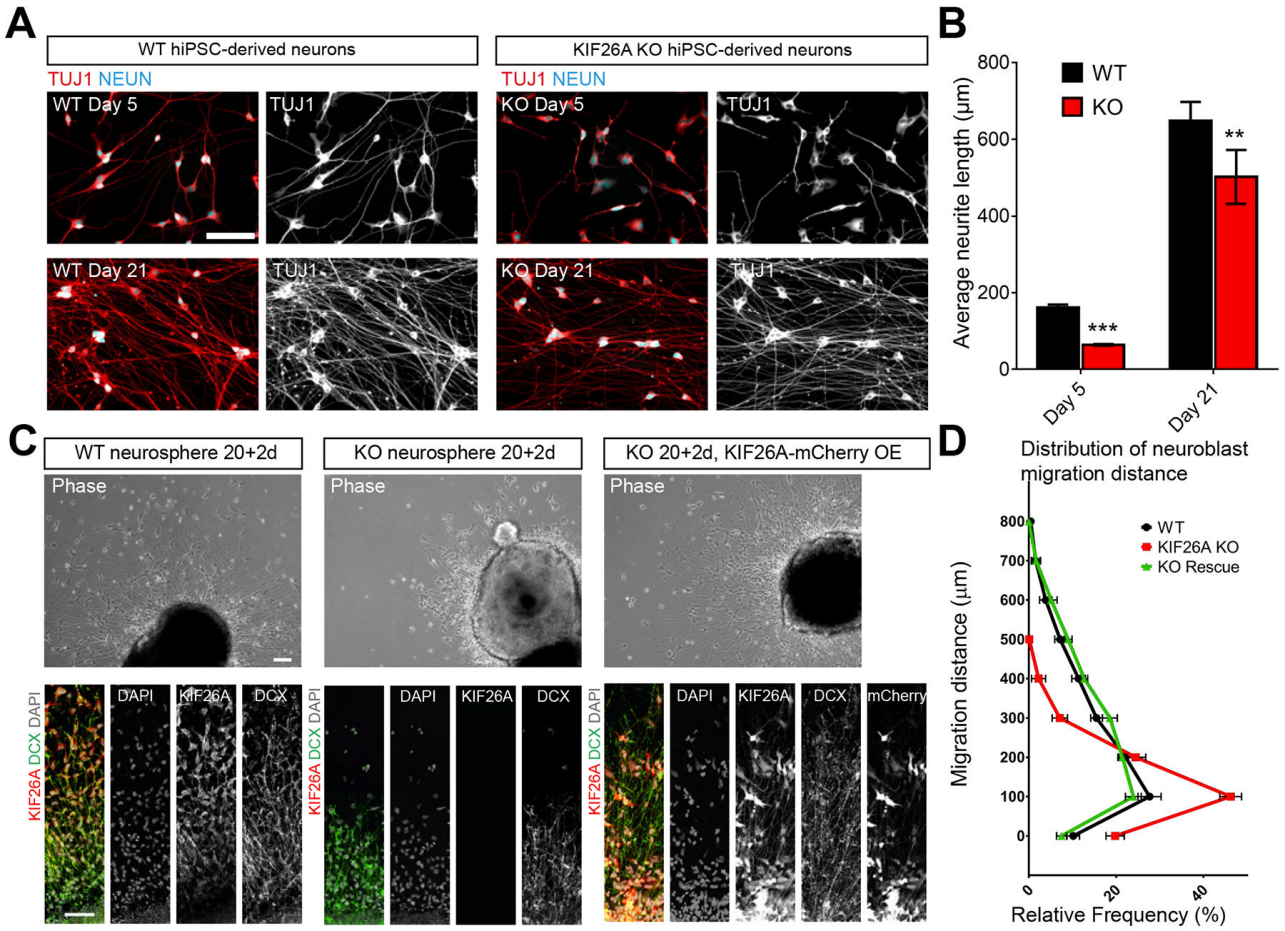
*KIF26A* KO Disrupts Neurite Outgrowth and Motility in Human iPSC-derived Neurons **(A)** Representative images of neurons differentiated from control (WT) and *KIF26A* KO (KO) hiPSCs at Day 5 and Day 21 showing shorter neurites of *KIF26A* KO neurons. Scale bar = 100μm. **(B)** Quantification of the average neurite length of WT and KO neurons shows reduced neurite outgrowth in *KIF26A* KO neurons. Average neurite length is calculated by dividing the total neurite length within a field of view to the number of NeuN^+^ neurons measured by an automated module. Values represent Mean± S.D. (n = 4 plates for WT, n = 8 plates for KO from two iPSC lines. Student’s t-test: **, p < 0.005; ***, p < 0.0005). **(C)** Phase contrast (top) and immunostaining (bottom) images of hiPSC-derived neurospheres 2 days after plating show reduced migration in *KIF26A* deficient neurons. Also shown on the right is KO neurosphere infected with lentivirus to express *KIF26A-mCherry* exogenously (rescue). Scale bars = 100μm (top), = 50μm (bottom). **(D)** Quantification of the distances migrating neuroblasts traveled away from the border of the neurosphere 2 days after plating. Values represent Mean± S.D. (n = 20 neurospheres from two pairs of WT and KO iPSC lines). See also Figure S5.

To investigate the impact of *KIF26A* loss in human neuronal development, we generated two pairs of isogenic homozygous *KIF26A* knockout (KO) human induced pluripotent stem cell (hiPSC) lines using CRISPR-Cas9 genomic editing (Figure S5). We differentiated *KIF26A* KO (referred to as KO) and the isogenic control (wild type, WT) iPSCs into cortical excitatory neurons. Automated high throughput imaging and machine learning-based neurite analysis revealed KO neurons showed shorter neurite length than WT neurons 5 days after plating, persisting to at least Day 21 (Figure 4A and B), consistent with the neuronal morphology defects observed in *Kif26a* KD mice (Figure 3G). Similar neurite outgrowth defects have previously been reported for *Kif26a* KO mouse enteric neurons, and were potentially associated with disrupted microtubule stability due to Kif26a loss (Zhou et al., 2009). We next differentiated iPSCs into forebrain neurospheres, and found that neuroblasts from the KO neurospheres migrated significantly shorter distances than control cells 2 days after attaching to a Matrigel-coated plate, and this defect could be rescued by restoration of *KIF26A* expression (Figure 4C and D).

### Human Forebrain Organoids Recapitulate the *in Vivo* Expression Specificity of KIF26A

**Figure 5.**
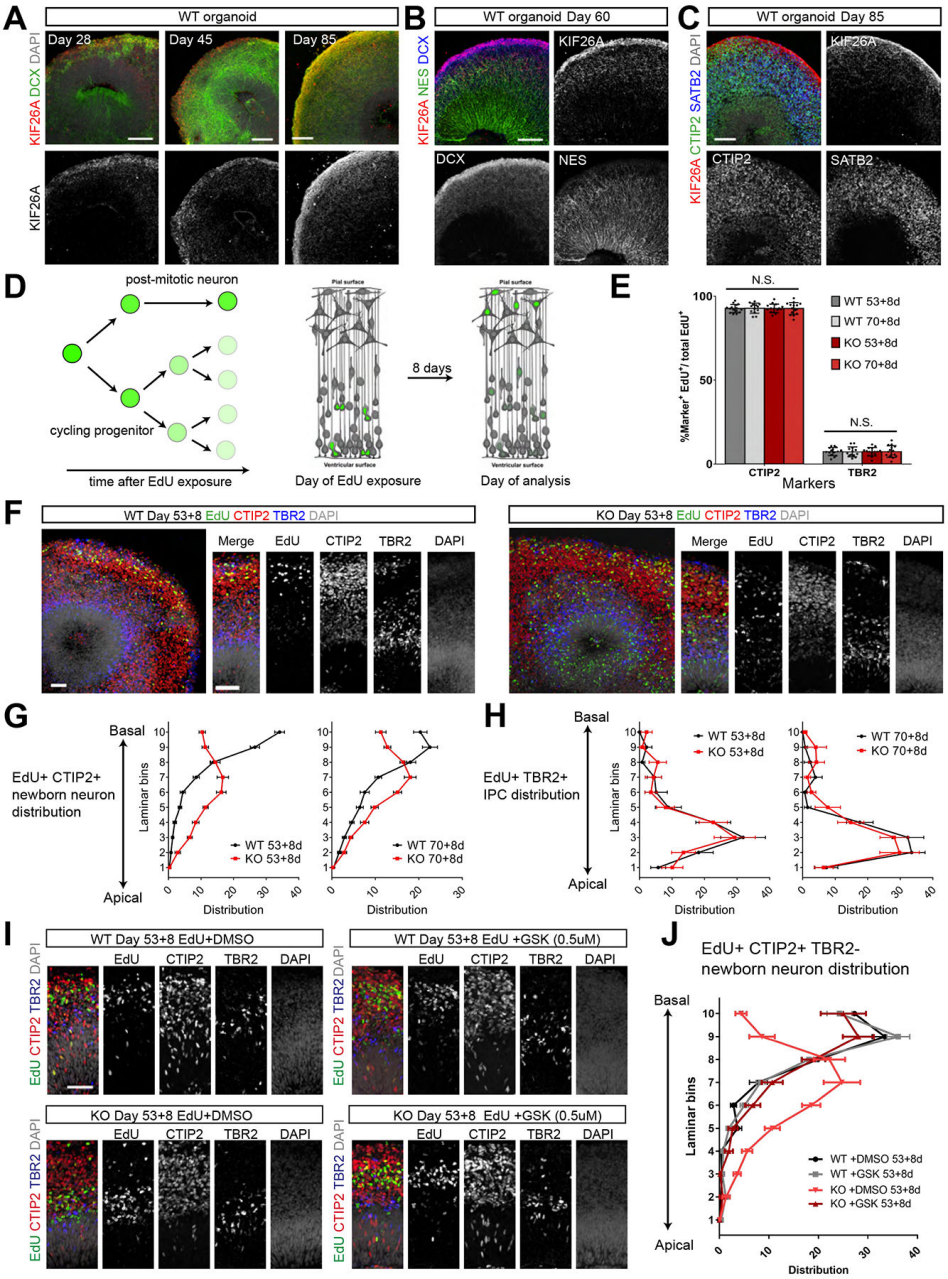
*KIF26A* KO Forebrain Organoids Exhibit Radial Migration Defects that Can Be Rescued by FAK Inhibition. **(A-C)** Representative images showing KIF26A is expressed by neurons but not progenitors in control forebrain organoids at various developmental stages. Scale bars = 100μm. **(D)** Schematic of the EdU pulse-chase strategy to track neuronal migration in forebrain organoids. After initial EdU exposure (1μM for 1hr), the EdU bond to DNA in dividing progenitors got diluted with each cell division. As a result, the cells displaying strong EdU detection intensity 8 days later were post-mitotic neurons born at the first cell division at the time of EdU exposure. Also see STAR Methods. **(E)** Quantification of the percentage of EdU-labeled cells expressing neuronal marker CTIP2 or IPC marker TBR2. Values represent Mean ± S.D. (n = 20 organoids, Student’s t-test: N.S., not significant). **(F)** Representative images showing the laminar distribution of EdU labeled cells in WT (left) and KO (right) forebrain organoids. Scale bars = 50μm. **(G)** Quantification of the laminar distribution of EdU-labeled CTIP2^+^ TBR2^−^ neurons in WT and KO forebrain organoids shows defective migration in *KIF26A* KO organoids. The cortical structure of organoids is divided evenly into 10 bins from basal (bin 10) to apical (bin 1) surfaces. Only Edu^+^, CTIP2^+^ and TBR2^−^ cells are counted to ensure counted cells are migrating neurons exclusively. Values represent Mean ± S.D. (n = 20 organoids from two pairs of isogenic lines). **(H)** Quantification of the laminar distribution of EdU-labeled TBR2^+^ CTIP2^−^ IPCs in WT and KO forebrain organoids shows normal IPC localization. Values represent Mean ± S.D., same samples as in **(G)**. **(I)** Representative images showing 8 days of 0.5μM FAK inhibitor GSK2256098(GSK) treatment rescued the laminar distribution of EdU-labeled neurons in KO organoids to resemble the localization in WT organoids. WT and KO forebrain organoids were treated with either DMSO or GSK during the 8-day chase period after EdU labeling at Day 53. Scale bar = 50μm. **(J)** Quantification of the laminar distribution of EdU-labeled CTIP2^+^ TBR2^−^ neurons in WT and KO forebrain organoids with and without GSK treatment. Values represent Mean ± S.D. (n = 10 organoids). See also Figures S6 and S7.

Next, we generated forebrain organoids from iPSCs using a revised feeder-free protocol (Figure S6A and B), and found that KIF26A expression in WT organoids recapitulates the pattern observed in human embryonic cortex. HiPSC-derived forebrain organoids produce cell types highly reminiscent of *in vivo* counterparts, and recapitulate the formation of an organized radial glia scaffold that supports the radial migration of excitatory neurons, offering a more representative model than monolayer or neurosphere neuronal cultures (Bershteyn et al., 2017; Lancaster and Knoblich, 2014; Pasca, 2018; Qian et al., 2018; Qian et al., 2016b; Qian et al., 2019; Qian et al., 2020). All tested WT and KO iPSC lines consistently differentiated into forebrain organoids with well-defined cortex-like cytoarchitectures forming CP-like, SVZ-like and VZ-like layers (referred to as CP, SVZ and VZ for short) (Figure S6C). Throughout various stages of WT forebrain organoid development, KIF26A was expressed specifically by excitatory neurons in the CP and SVZ, co-localizing with neuronal markers DCX, CTIP2, SATB2 (shown by immunostaining), and *RORB*, *RBFOX3* (NeuN) (shown by ISH) (Figures 5A, B, and S6D and E), faithfully recapitulating the expression pattern in the developing human cortex. Neurogenesis of early-born (CTIP2^+^) and late-born excitatory neurons (SATB2^+^) appeared normal in KO organoids, and KIF26A expression did not display neuronal subtype preferences (Figures 5C and S6F). We separately characterized the proliferation of SOX2^+^ TBR2^−^ radial glia and TBR2^+^ IPC in KO organoids and did not find significant changes in the abundance of dividing cells and the division angle of ventricular radial glia (Figure S6G-I).

### KIF26A Regulates Neuronal Radial Migration in Forebrain Organoids via FAK signaling

We analyzed radial migration of new-born neurons in WT and KO organoids by EdU (5-ethynyl-2'-deoxyuridine) birth-dating and saw a striking neuronal radial migration defect. We pulsed organoids with EdU at Day 53 and Day 70, and detected EdU-labeled cells after an 8-day chase period (STAR Methods). Because EdU taken up by a dividing cell is diluted each time a progenitor cell divides, only postmitotic neurons born at the time of EdU exposure show high fluorescence intensity 8 days later, allowing us to track neurons born at a known birth-date and measure the distance they migrated (Figure 5D). Quantitative analysis showed that in both WT and KO organoids, the majority of EdU-labeled cells 8 days post exposure are CTIP2^+^ TBR2^−^ excitatory neurons (~93%), while the rest are TBR2^+^ CTIP2^−^ IPCs (~7%). This confirmed that neuronal differentiation was unperturbed in KO organoids (Figure 5E). In WT organoids, KIF26A was expressed in EdU-labeled neurons, but the expression was completely abolished in KO organoids (Figure S7A and B). While most EdU-labeled CTIP2^+^ TBR2^−^ neurons in WT organoids migrated to the upper layer of the CP after 8 days, many labeled neurons in the KO organoids were found in the SVZ, suggesting stalled or arrested radial migration (Figures 5F and G, and S7C), consistent with the defect observed in *Kif26a* KD mouse brains. This migratory deficit is specific to neurons, because EdU-labeled TBR2^+^ CTIP2^−^ IPCs showed the same laminar localization in KO organoids as in WT (Figure 5H). KIF26A’s known binding target, FAK, has been reported to specifically regulate excitatory neuron radial migration but not interneuron tangential migration, and both loss and overexpression of FAK led to arrest of excitatory neurons in the mouse cortex (An et al., 2018; Valiente et al., 2011; Wang et al., 2018; Yokota et al., 2007). Consistent with KIF26A’s inhibition of FAK phosphorylation (Wang et al., 2018), phospho-FAK was more abundant in the CP of KO organoids than WT (Figure S7D). Remarkably, when we treated WT and KO organoids during the EdU chase period with a selective FAK inhibitor, GSK2256098 (GSK), the localization of EdU-labeled neurons in KO organoids were restored to the CP, resembling WT organoids (Figure 5I and J). The rescue effect of FAK inhibition strongly suggests that KIF26A regulates radial migration via a FAK-dependent mechanism.

### *KIF26A* KO Organoids Show Aberrant Lamination and Elevated Apoptotic Cell Death

**Figure 6.**
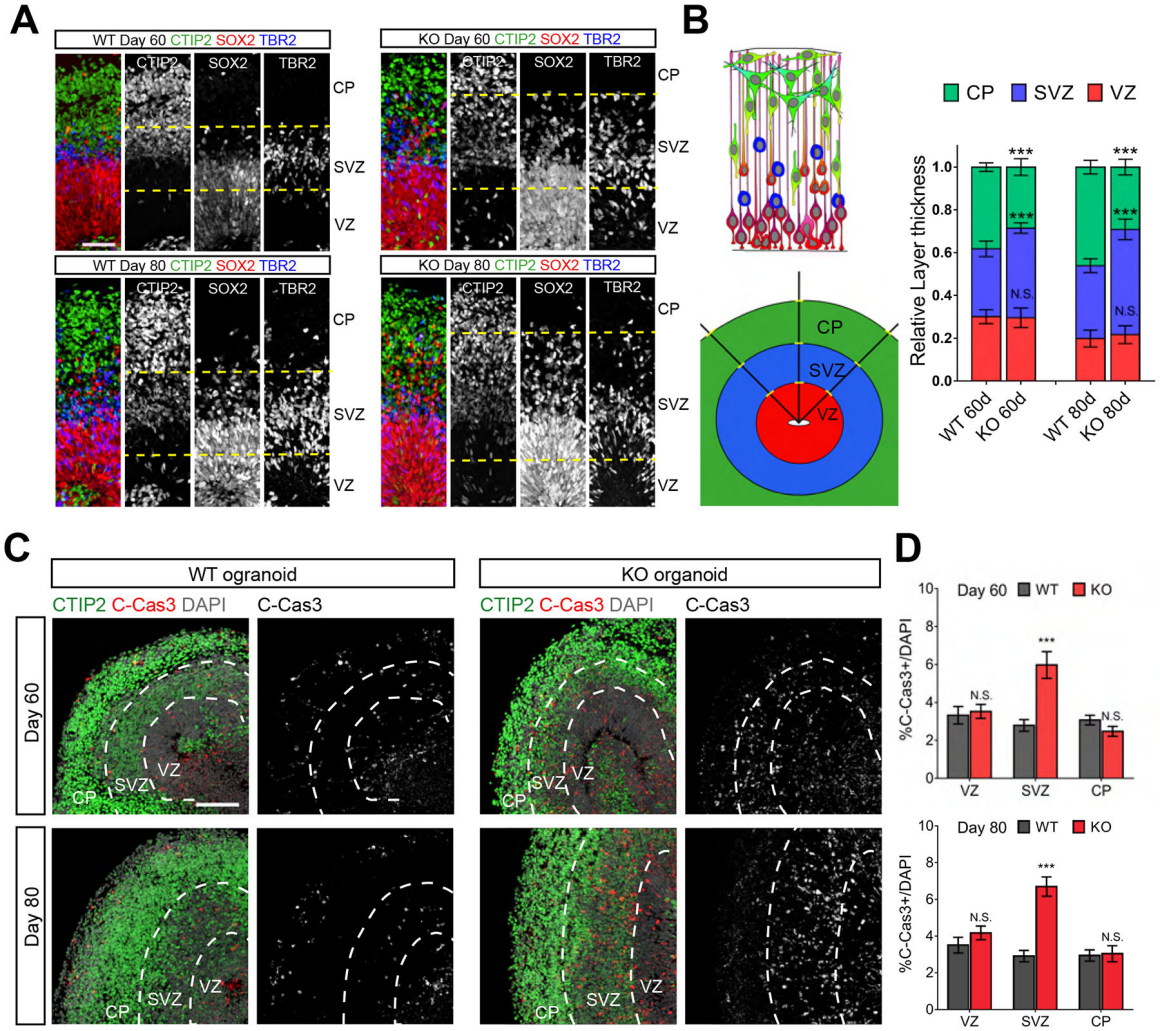
*KIF26A* KO Forebrain Organoids Exhibit Altered Lamination and Elevated Apoptotic Cell Death. **(A)** Representative images showing the defective laminar organization in *KIF26A* KO forebrain organoids compared to WT. Dashed lines help to illustrate the borders between CP, SVZ and VZ. Scale bars = 50μm. **(B)** Quantitation of relative layer thickness in WT and KO organoids shows enlarged SVZ and reduced CP in *KIF26A* KO organoids. Left, schematics showing the measurement of layer thickness. For each cortical structure, three measurements were taken at 45° angle pointing towards the basal surface and taken average for the quantification. Values represent Mean ± S.D. (n=20 organoids from two pair of isogenic lines. Student’s t-test: N.S., not significant; ***, p < 0.0005). **(C)** KO organoids show elevated apoptosis at Day 60 and 80. Dashed lines delineate the borders between the CP, SVZ and VZ. Scale bar = 100μm. **(D)** Quantification of the density of apoptotic cells in the VZ, SVZ and CP layers of WT and KO organoids. Values represent Mean ± S.D. (n = 20 organoids from two pairs of isogenic lines. Student’s t-test, ***, p < 0.0005; N.S., no significant difference). See also Figure S7.

Measuring the relative thickness of VZ, SVZ and CP revealed a significantly expanded SVZ and thinner CP in KO organoids compared to WT at both Day 60 and Day 80 (Figure 6A and B), likely due to arrested migratory neurons failing to reach the CP and overpopulating the SVZ. Since brain atrophy (identified in patient A01 with a frameshift mutation) is often associated with neuronal death (Little and Dwyer, 2019; Poulton et al., 2011; Zhang et al., 2014), we compared the abundance of apoptotic cell death in WT and KO forebrain organoids and found increased apoptosis in KO organoids at both Day 60 and 80 (Figure 6C). The increase in apoptotic cells in KO organoids was specifically limited to the SVZ, suggesting that the apoptotic cells included migratory neurons (Figure 6D), although the identities of cleaved-caspase 3^+^ cells could not be determined due to the loss of marker expression after cells commit to apoptosis. While FAK inhibition with GSK rescued the migratory defect, apoptosis remained high in GSK-treated KO organoids, suggesting that KIF26A regulates apoptosis independent of FAK (Figure S7E and F).

### Single-cell RNA-seq Reveals Cell Type-Specific Transcriptional Changes in KO Organoids

**Figure 7.**
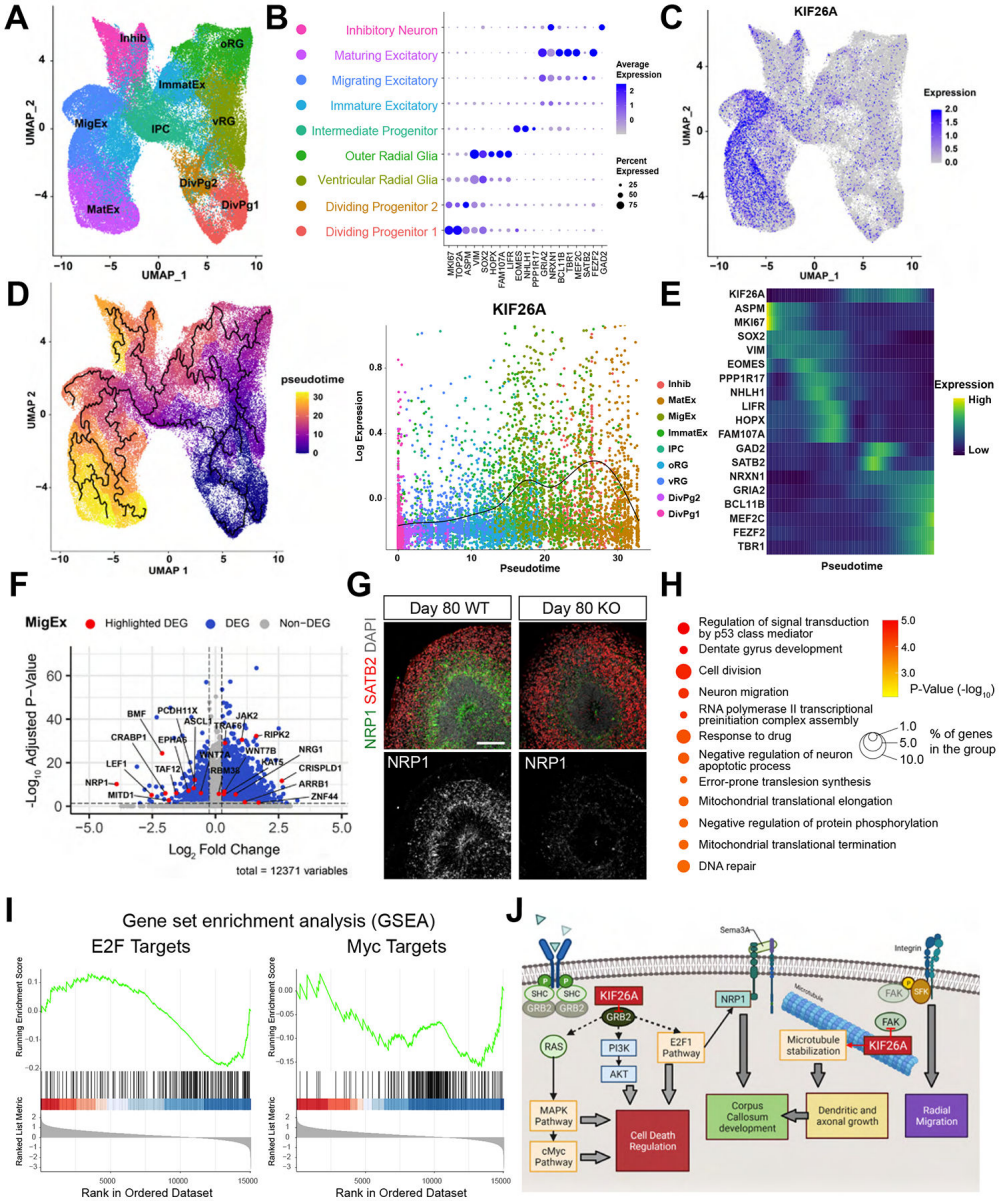
Loss of *KIF26A* Leads to Transcriptional Changes in Forebrain Organoids. **(A)** Graph-based clustering of single cells from WT and KO brain organoids at Day 60 and Day 90 (n=46,776 cells for WT, n= 36,645 cells for KO). DivPg, dividing progenitors; vRG and oRG, ventricular and outer radial glia; IPC, intermediate progenitor cells; Inhib, inhibitory neurons; ImmatEx, immature excitatory neurons; MigEx, migrating excitatory neurons; MatEx, maturing excitatory neurons. **(B)** The expression of selected well-known marker genes used for cell type classification. **(C)** UMAP feature plot showing *KIF26A* expression is enriched in MigEx and MatEx clusters. **(D)** Pseudotime analysis showing the developmental trajectory (left) calculated using Monocle 3. Right, *KIF26A* expression ordered by pseudotime. Dots represent individual cells colored based on cluster. Trend-line shows *KIF26A* expression as function of pseudotime, calculated by fitting a quasipoisson model to the data. **(E)** Expression of well-known marker genes across the pseudotime trajectory. **(F)** Volcano plot showing differentially expressed genes (DEGs) between WT and KO cells in migrating excitatory neurons. Significant DEGs with adjusted p-value < 0.05, and Log_2_ fold change > 0.25 or < −0.25, are shown in blue. Selected DEGs involved in neuronal survival and apoptosis are highlighed in red. **(G)** Immunostaining validating decreased NRP1 expression in KO organoid. Scale bar = 100μm. **(H)** Gene ontology (GO) analysis (FDR<0.05) of 348 significantly downregulated (adjusted p-value< 0.05, Log_2_ fold change < −1) genes in KO migrating excitatory neurons compared to WT. Size and color of the bubbles represent the proportion of commonly dysregulated genes enriched in each pathway and the significance of enrichment, respectively. **(I)** Gene set enrichment analysis (GSEA) enrichment score curves showing downregulation of E2F and Myc pathways in KO migrating and maturing excitatory neurons compared to WT. Hallmark Gene Sets from MSigDB Collections were included for the analysis. **(J)** Schematic illustration of proposed molecular mechanisms, by which KIF26A modulates radial migration, dendritic and axonal development and apoptosis in excitatory neurons. See also Figure S8.

We performed single-cell RNA-seq (scRNAseq) at Day 60 and 90 to compare WT and KO organoids, and discovered transcriptional changes potentially underlying increased apoptosis in the absence of *KIF26A*. After quality control filtering, we identified 9 distinct cell clusters annotated based on expression of well-known cell-type markers (Girskis et al., 2021; Polioudakis et al., 2019; Pollen et al., 2015), including dividing progenitor cells, outer and ventricular radial glia, intermediate progenitors, inhibitory neurons and three groups of cortical excitatory neurons of different maturation stages (Figure 7A and B). The fidelity of cell-type classification was validated by the remarkable agreement when comparing organoid datasets to a compiled fetal human cortex atlas (Figure S8A and B). The cells from WT and KO organoids integrated well without noticeable segregation (Figure S8C), and each individual organoid analyzed from both WT and KO lines contained all cell types at similar proportions, indicating high reproducibility of our organoid differentiation, and confirming our earlier finding that *KIF26A* loss does not perturb progenitor differentiation or alter cell-type composition (Figure S8D). Expression of *KIF26A* was specifically enriched in Migrating Excitatory Neuron (MigEx) and Maturing Excitatory Neuron (MatEx) clusters (Figure 7C), consistent with the cell type-specificity in human fetal cortex (Figure 2B). Pseudotime analysis revealed MigEx cells were at an earlier maturation stage than MatEx, and MigEx had the strongest enrichment of *KIF26A* expression (Figure 7D and E), supporting *KIF26A*’s role in the radial migration of excitatory neurons.

Because our data strongly support cell-autonomous mechanisms acting specifically on excitatory neurons, we focused on the two *KIF26A* expressing clusters (MigEx and MatEx) for further analysis. Differential Expression Analysis (DEA) between KO and WT cells identified differentially expressed genes (DEGs) in the two clusters. *KIF26A* KO cells showed downregulation of several known regulatory genes involved in neuronal apoptosis, such as *NRP1, PARP2, BMF* and *TAF12* and upregulated genes included many genes involved in apoptosis and the MAPK pathway, such as *MAPK14, JAK2, ARRB1, RIPK2* and *TRAF7*, consistent with the previous report that KIF26A suppresses the MAPK pathway via inhibition of GRB2-mediated signaling (Ma et al., 2021; Zhou et al., 2009) (Figures 7F, and S8E and F). Immunostaining for NRP1 (neuropilin-1) showed exclusive expression in the SVZ, and validated its downregulation in KO organoids (Figure 7G). Top gene ontology (GO) terms of significantly downregulated genes in MigEx included regulation of ‘p53 signal transduction’ and ‘negative regulation of neuron apoptotic process’ (Figure 7H). Lastly, we performed gene set enrichment analysis (GSEA) between KO and WT cells in the MigEx and MatEx clusters. Among all 50 hallmark gene sets (Liberzon et al., 2015), G2M checkpoint, MYC Targets and E2F Targets showed significant downregulation, and network analysis revealed shared DEGs between the three pathways (Figures 7I and S8G). Importantly, both MYC and E2F pathways are critical regulators of transcription and cell survival, and are regulated directly and indirectly by signal transduction adaptor protein GRB2, which is inhibited by KIF26A via direct binding (Chaussepied and Ginsberg, 2004; Zhang and Liu, 2002; Zhou et al., 2009).

## DISCUSSION

Combining human genetics with *in vivo* and *in vitro* models for functional characterization, we discovered that *KIF26A* regulates radial migration, dendritic and axonal development and apoptosis in excitatory neurons, and is critical to normal corticogenesis and corpus callosum development. Because we first established that the missense variants identified in our patients cause at least partial loss of KIF26A function, our LoF experiments offer direct insights into the etiology underlying the patients’ developmental abnormalities. Our KO organoid experiments are especially analogous to Patient A01, who carries a homozygous frameshift mutation that completely abolishes *KIF26A* function. The association between *KIF26A* and the aforementioned developmental processes is strengthened by the high coherence in the patients’ phenotypic presentations. Abnormalities characterized by dilated ventricles and reduced white matter, associated with axonal developmental defects, are seen in all 4 patients in which axonal patterns could be studied. Cortical malformations including microcephaly, polymicrogyria and schizencephaly (Bahi-Buisson et al., 2014; Moffat et al., 2015; Smith et al., 2018), are seen in 4 of the 5 patients. Defective CC development is seen in all 4 patients for whom adequate MRI data were available to evaluate the CC.

Although many kinesins have been associated with neurodevelopmental disorders, KIF26A is unique because it does not function as a molecular motor, but instead modulates signal transduction via direct binding to FAK and GRB2 (Wang et al., 2018; Zhou et al., 2009). The remarkable cell-type and temporal expression specificity of KIF26A also distinguishes it from most other kinesins with characterized functions in the CNS (Konjikusic et al., 2021), highlighting its critical requirement specifically for embryonic brain development. We propose a model in which KIF26A (1) regulates dendritic and axonal growth as a microtubule stabilizer, and thus regulates white matter and CC development; (2) regulates radial migration and terminal localization via FAK inhibition; (3) and regulates neuronal apoptosis by inhibiting GRB2-mediated signaling pathways (Figure 7J).

One significant advantage of our study is the multipronged approach using multiple model systems to obtain coherent functional discovery, which synergistically strengthens the plausibility of our conclusions. The defects in dendritic and axonal development in mice echoes our finding that the KO human iPSC-derived neurons had shorter neurites. Similarly, *KIF26A* loss perturbed radial migration and localization of excitatory neurons in both mouse and human organoids, which can be pharmacologically rescued by selective inhibition of FAK. While we only observed increased apoptosis in *KIF26A* KO organoids, but not in electroporated mouse brains, one explanation for this difference is that KO organoids model complete LoF, similar to Patient A01 with a homozygous frameshift, who is the only patient diagnosed with cerebral atrophy, which typically reflects increased apoptosis. In contrast, incomplete KD induced by shRNA IUE in the mouse brain did not cause dramatic increase in apoptosis despite the neuronal mis-localization, recapitulating the partial LoF in patients carrying biallelic missense mutations. An alternative interpretation is that KIF26A’s regulation of neuronal apoptosis is a function specific to humans, but not mice. While we do not have sufficient evidence to support this interpretation, we resorted to scRNAseq comparison in human organoids to investigate the pathways and genes affected by *KIF26A* loss specific to human cortical development.

Our scRNAseq analysis offers molecular insights into the unexpected transcriptional regulatory functions of *KIF26A*, which may underlie the apoptosis phenotype. GO analysis and GSEA revealed disruption of MAPK, E2F and MYC pathways, echoing two very recent studies also reporting KIF26A’s involvement in MYC and E2F pathways in cancer (Ma et al., 2021; Xu et al., 2020). We propose that KIF26A’s inhibition of GRB2-dependent growth factor signaling modulates the MAPK and E2F pathways, either directly or indirectly, and subsequently the MYC pathway, which may explain the transcriptional changes that cause elevated apoptosis in KO organoids (Figure 7J) (Wang et al., 2007a; Zhou et al., 2009; Zhu et al., 2008). Notably, *NRP1*, the most strongly downregulated gene in the KO MigEx cluster, has been reported to be a regulator of p53-transduced signals and is crucially required for CC development (Jiang et al., 2007; Piper et al., 2009; Sun et al., 2017; Wang et al., 2007b). NRP1 is the receptor for the critical callosal axonal guidance molecules SEMA3A and SEMA3C (Niquille et al., 2009; Zhao et al., 2011), and loss of NRP1 has been shown to impede callosal axon growth cell-autonomously and disrupt CC formation (Edwards et al., 2014; Piper et al., 2009). Moreover, NRP1 is a direct target regulated by the E2F pathway, the most significantly downregulated pathway in our KO organoids (Figure 7I)(Jiang et al., 2007). Therefore, we propose that KIF26A is required for callosal axon development by a combinatorial mechanism of regulating microtubule stability and modulating the expression of NRP1 (Figure 7J). Taken together, our findings on the non-motor functions of KIF26A reveal a greater versatility of functions within the kinesin superfamily, and underscore their roles in development and diseases.

### Limitations of the study

One limitation of our study is that the mouse and human organoid experiments modeled *KIF26A* loss instead of directing characterizing the pathogenic effects of the missense variants discovered in the patients, because we do not have access to patient-derived iPSCs. To circumvent this limitation, we combined patient genetics analysis and *in vitro* cellular assay to establish that the pathogenicity of the missense variants is due to LoF, and then focused on characterizing the developmental impairment caused by *KIF26A* loss. Another limitation is that we could not directly illustrate the molecular pathways via which GRB2-mediated signaling is responsible for the gene expression changes and elevated cell death in KO organoids. Our current study provides indirect evidence from scRNAseq comparison in KO and WT organoids, which showed dysregulation of GRB2-mediated pathways including MAPK, MYC and E2F. While our study has illustrated that *KIF26A* loss underlies these dysregulations, in-depth biochemistry in more simplistic models to characterize the detailed molecular changes upon *KIF26A* loss is a direction for follow-up investigation.

## STAR METHODS

### RESOURCE AVAILABILITY

#### Lead Contact

Please contact Dr. Christopher A. Walsh at Christopher.Walsh@childrens.harvard.edu if you would like to request materials used in this study.

#### Material Availability

This study did not generate any new reagents or tools.

#### Data and Code Availability

- Single-cell RNA-seq data have been deposited at GEO and are publicly available as of the date of publication. Accession number is listed in the key resources table.
- Computer codes and algorithms used in this study are available from the lead contact, Dr. Christopher A. Walsh (Christopher.Walsh@childrens.harvard.edu) upon reasonable request.
- Any additional data and information that support the findings of this study are available from the lead contact Dr. Christopher A. Walsh upon reasonable request.

### EXPERIMENTAL MODEL AND SUBJECT DETAILS

#### Human subjects and samples

Research performed on samples of human origin was conducted according to protocols approved by the institutional review boards of Boston Children’s Hospital, Beth Israel Deaconess Medical Center, Montreal Neurological Institute, University of California San Francisco, Women’s and Children’s Hospital in Adelaide, Private Reyap Istanbul Hospital and Baylor College of Medicine. Subjects were identified and evaluated in a clinical setting, and biological samples were collected after obtaining written informed consent.

Fetal brain tissue was received after release from clinical pathology, with a maximum post-mortem interval of 4 h. Samples with known anomalies were excluded. Tissue was transported in Hibernate-E media on ice to the laboratory for research processing. Following fixation (4% PFA in PBS for 24 hours), tissue was prepped for paraffin sectioning. Coronal paraffin sections (5 μm) were generated from a gestation week 22 medial cerebral cortex sample, and deparaffinized following standard Formalin-Fixed Paraffin-Embedded (FFPE) sample preparation and pretreatment protocol per manufacturers guidelines (ACD Document, 322452).

#### Phenotypic assessment

All affected individuals and clinical data were examined by neurologists and radiologists, and malformations were diagnosed using accepted clinical criteria. Clinical descriptions of the families were based on review of primary clinical records, and report of clinicians who directly examined the patients. Records were reviewed by at least two members of the research team with clinical backgrounds, and phenotypic comparisons were performed after patient records were summarized into a phenotype table.

#### Additional Clinical Descriptions of Patients Presented:

A01, the proband of Family A (BAB10995), presented with microcephaly (−3.45SD) and an MRI suggesting a component of cerebral atrophy, as well as dysmorphic features, ileus with megacolon. Brain MRI at 3m of age showed reduced white matter and a thin corpus callosum (Figure 1Aa-b). This consanguineous family had six other children who died in infancy, but on whom clinical details are sparse and genetic testing not available. Exome sequencing revealed inherited bi-allelic frameshift variants in *KIF26A* (p.Ala1148Cfs*20). Additional siblings who died in early life included: (1) a male sibling who died at 1.5 month without phenotyping, (2) a female sibling who died at 3 month with presumed ileus, (3) a male sibling who died at 4 month with ileus, hydrocephalus and hypotonia, (4) a female sibling who died at 1 year with no clinical information, (5) a male sibling who died at 2 month with esophagus atresia, esophagus trachea fistula, anus atresia/fistula, Dandy walker malformation and congenital hypothyroidism hypospadias, (6) a male sibling who died at birth with no clinical information.

B01, identified from Family B (PED026C), was diagnosed prenatally with bi-lateral schizencephaly at 21 weeks gestation. Other abnormalities included high arched palate and long philtrum, identified on fetopsy after termination. This patient was also noted to have an absent stomach bubble on prenatal imaging. Fetopsy MRI confirmed the bilateral schizencephaly and further revealed abnormal grey-white matter differentiation, with an abnormally fused molecular layer (though too early in gestation to call polymicrogyria specifically) (Figure 1Ac-d). Sequencing for COL4A1 and COL4A2 variants was normal, and WES revealed inherited compound heterozygous variants: p.Arg721Cys, and p.Ala1559Val.

C01, identified from Family C (SHE_1305), is a male born to non-consanguineous parents at 41 weeks via emergency c-section after a pregnancy complicated by borderline gestational diabetes. He experienced fetal distress and ingested meconium during the end of labor and delivery, though the nursery course was not complicated. SHE_1305 has mild developmental delay, pulling to stand at 12 months, and walking up/downstairs at 24 months. He is diagnosed with a learning disability, and is enrolled in partial IEP support including speech therapy. His parents reported issues with processing, learning, and behavior (with impulsivity) while in grade school. No additional medical complications were identified. A brain MRI at 18 years demonstrated complete agenesis of the corpus callosum, with imaging also detecting enlarged anterior commissure, absent hippocampal commissure, mild colpocephaly, mildly decreased white matter volume, and present Probst Bundles.

D01, identified from Family D (PMG_14800), was referred to our laboratory with a diagnosis of polymicrogyria and hydrocephalus, tonic-clonic seizures since 1 year of age, on medication, divergent left strabismus and bilateral nystagmus. The MRI images and further clinical information were not available for review.

E01, the proband of Family E (BAB5949), is a male born to consanguineous parents from Turkey by C-section at 38 weeks. His delivery was complicated by meconium aspiration. His birth weight and length were 2.73 kg and 45 cm. He was evaluated at 3 years 6 months of age due to growth retardation and developmental delay. He exhibited hypotonia and dysmorphic facial features including synophrys and brachydactyly leading to concern for Cornelia de Lange syndrome (CdLS). Brain MRI demonstrated thin corpus callosum, mild ventriculomegaly and polymicrogyria. Exome sequencing demonstrated homozygous KIF26A: c.4804C>T; p.Arg1602Trp. No variants in known CdLS genes or other known or candidate neurodevelopmental disease genes were identified.

#### N2A, HEK293T and SH-SY5Y cell lines

All cell cultures were maintained in 5% CO_2_ incubator at 37 °C. Neuro2A/N2A cells (ATCC, CCL-131), HEK293T (ATCC, CRL-3216) and SH-SY5Y (ATCC, CRL-2266) were grown in 10% fetal bovine serum, Dulbecco’s Modified Eagle Medium, and 1X Penicillin-Streptomycin.

#### Generation and characterization of iPSCs and KIF26A KO iPSCs

The PGP1 and 280 human iPSC lines were previously generated and fully characterized(Lee et al., 2009; Schlaeger et al., 2015). Both iPSC lines were reprogrammed from primary fibroblast from healthy donor using CytoTune^™^-iPS 2.0 Sendai Reprogramming Kit (ThermoFisher). Karyotyping analysis was performed by commercial services KaryoStat (ThermoFisher) and G-Banding (Cell Line Genetics).

CRISPR-Cas9-mediated KIF26A KO in PGP1 and 280 iPSC lines was performed by Synthego (Redwood City, CA). Briefly, guide RNAs was designed to create a frame-shift insertion at the fourth (for PGP1) and third (for 280) exon of *KIF26A* (Chr14: 104,624,154 and Chr14:104, 618, 554) and maximize the number of mismatches with other sequences in the genome to reduce off-target effects. Guide RNAs (CGGCCCUGAUGGCUUGUCGA for PGP1; CCAGCACCACGACCAGCUCG for 280) were synthesized and complexed with SpCas9 to form a ribonucleoprotein (RNP). RNP were transfected into the iPSCs via electroporation together with positive control sgRNA (RELA). Transfected cell pool was analyzed with Sanger sequencing to confirm successfully edits. The edited pool is used to seed single cells for clonal expansion. Each well seeded is imaged every 2-3 days and tracked to ensure the population is truly clonal and only the progeny of a single cell. Two clones were selected and passed characterization of genotyping with sanger sequencing, karyotyping by KaryoStat, viability test, mycoplasma test and pluripotency tests.

#### Maintenance of iPSCs

Feeder-free human iPSCs were cultured in mTeSR Plus medium supplemented with 1X Penicillin/Streptomycin. Culture medium was changed daily. Human iPSCs were passaged weekly onto a new plate coated with Matrigel. iPSCs were detached from the plate by treatment of ReLeSR enzyme-free passaging reagent following the manufacturer’s instructions. Cells were routinely examined for mycoplasma and karyotype abnormalities with KaryoStat. The iPSCs used throughout the study were below passage 50. All studies were performed with approved protocols of Boston Children’s Hospital.

#### Mice

Timed pregnant mice (*Mus musculus,* CD1 strain) were obtained from Charles River Laboratories between 9-11 gestational days, prior to performing the *in utero* electroporation procedure. All experiments were reviewed and approved by the Institutional Animal Care and Use Committee (IACUC) at Boston Children’s Hospital (BCH). Animals were housed in BCH facilities and were maintained and cared for according to BCH and NIH animal research guidelines.

### METHOD DETAILS

#### Human genetics

DNA derived from peripheral blood in PMG14801 and their parents (Family D); SHE_1305 and their parents (Family C); PED026C (blood and lung tissue in proband), their parents, and unaffected siblings (Family B); BAB10995, their parents, and unaffected siblings (Family A); BAB5949 and their mother were analyzed by Whole Exome Sequencing (WES). Subjects were identified and evaluated in clinical settings, and biological samples were collected for research purposes after obtaining written consent. These studies were approved by the necessary institutional review boards; PMG14801 (Family D), Boston Children’s Hospital and Montreal Neurological Institute; SHE_1305 (Family C), University of California San Francisco; PED026C (Family B), Women’s and Children’s Hospital in Adelaide (IRB number: HREC15/WCHN/35); BAB10995 (Family A) and BAB5949 (Family E), Baylor College of Medicine (IRB approval number: H-29697). The cases in this cohort were ascertained and processed using a variety of different methods but connections were made using MatchMaker Exchange.

WES and data processing for PMG21801, SHE_1305 and PED026C was performed by the Genomics Platform at the Broad Institute of Harvard and MIT (Cambridge, MA, USA). Whole exome sequencing was done on DNA samples (>250 ng of DNA, at >2 ng/μL) using Illumina exome capture (38 Mb target). The exome-sequencing pipeline included sample plating, library preparation (2-plexing of samples per hybridization), hybrid capture, sequencing (150 bp paired reads), sample identification QC check, and data storage. The hybrid selection libraries cover >90% of targets at 20x and a mean target coverage of ~100x. The exome sequencing data was de-multiplexed and each sample’s sequence data were aggregated into a single Picard BAM file. Exome sequencing data was processed through a pipeline based on Picard, using base quality score recalibration and local realignment at known indels. The BWA aligner was used for mapping reads to the human genome build 37 (hg19). Single Nucleotide Polymorphism (SNPs) and insertions/deletions (indels) were jointly called across all samples using Genome Analysis Toolkit (GATK) HaplotypeCaller package version 3.4. Default filters were applied to SNP and indel calls using the GATK Variant Quality Score Recalibration (VQSR) approach. Lastly, the variants were annotated using Variant Effect Predictor (VEP). For additional information please refer to Supplementary Section 1 of the paper describing ExAC (Lek et al., 2016). The variant call set was uploaded on to Seqr and analysis was performed using the various inheritance patterns. Candidate variants were validated further by Sanger sequencing. Segregation analysis was performed on unaffected siblings using Sanger sequencing.

For BAB10995 and BAB5949, and their family, next-generation, massively parallel Exome sequencing (ES) and whole-genome sequencing (WGS) were performed at the Human Genome Sequencing Center (HGSC) at BCM through the Baylor-Hopkins Center for Mendelian Genomics initiative.24 For exome sequencing after quality control (QC), libraries were prepared with various methods over a span of time. Each new method was validated prior to implementation. Pre-capture libraries were prepared using Phusion or KAPA Hyper reagents, then pooled into 4-plex library pools and hybridized in solution to the HGSC-designed Core capture reagent 29 (52 Mb, NimbleGen) or 6–10-plex library pools that used the custom VCRome 2.1 plus custom Spike-In design (42Mb, NimbleGen) according to the manufacturer’s specifications, with minor revisions. Paired-end sequencing was performed for all samples in a format of multiplexed pools to generate an average depth of coverage of 1193 using the Illumina HiSeq2000 or NovaSeq6000 instrument. With an average sequencing yield of 11.5 Gb, the samples achieved 97% of the bases covered to a depth of 203 or greater.

#### Identification and segregation of alleles

Candidate variants identified via WES were evaluated via manual review by research team for their rarity, predicted effects, population frequencies, and review of literature. For each family, all candidate variants were ruled out except *KIF26A*, and following prioritization of *KIF26A* as a candidate gene for each family, Sanger confirmation of each variant was performed on original patient samples. Sanger confirmation of variants was also performed on first degree relative samples available to confirm segregation of alleles with phenotype. Families with other high priority candidate genes, or in which *KIF26A* variants did not Sanger validate, or failed to segregate with the neurological phenotypes, were excluded from further analysis.

#### KIF26A protein architecture prediction

The human KIF26A sequence was retrieved from UniProt (https://www.uniprot.org/uniprot/Q9ULI4) and its predicted secondary structure by PsiPRED was plotted by REPPER at the MPI Bioinformatics Toolkit (Zimmermann et al., 2018), showing the distribution of high-confidence alpha-helix and beta-strand (red and green peaks, respectively) along the chain. The Metapredict server (https://metapredict.net/) was used to plot contrasting predictions of disorder and AlphaFold2 structural confidence scores (red and blue peaks, respectively), the latter a strong indicator of underlying globular structure (Emenecker et al., 2021). These predictions were used to locate the folded protein domains D1-3 in KIF26A, that were then mapped to the calculated AlphaFold database model of human KIF26A, ID # AF-Q9ULI4-F1 for fold analysis (Tunyasuvunakool et al., 2021).

#### HEK293T cell culture, immunostaining and Western Blot

HEK293T cells were grown as adherent cultures to 40% confluency in 12-well cell culture plates. Empty mCherry plasmid, mCherry-tagged WT human KIF26A and KIF26A patient variants expression plasmids were transfected with Lipofectatmine 3000 reagent. Because HEK293T cells have almost no base-line expression of KIF26A, the KIF26A expression detected in immunostaining and Western Blot is due to the expression of transfected constructs. For evaluation of expression and intracellular localization of WT and variant KIF26As, the cells were permeabilized with 0.5% Triton-X in PBS for 30 min and blocked with blocking solution of 10% donkey serum in PBS and 0.05% Triton-X for 15 min. Primary antibodies diluted in blocking solution were applied to the sections in room temperature for 2 hrs. The primary antibodies used and their dilution are summarized in the Key Resource Table. After washing with PBST for a minimum of 3 times, secondary antibodies and DAPI (1:2000) diluted in blocking solution were applied to the sections for 1 hour at room temperature. Secondary anti-bodies were: AlexaFluor 488, 555, 594, or 647 -conjugated donkey antibodies (Invitrogen) used at 1:500 dilution. Finally, cells were washed with PBST for a minimum of 3 times before mounting with Vectashield Vibrance Antifade Mounting Medium.

Western Blot was performed to determine the expression level of KIF26A. 48 hours post-transfection cells were harvested and protein extraction was performed using RIPA Lysis and Extraction Buffer following the manufacturer’s manual. Lysates were loaded into 4-20% Mini-Protean TGX Precast Protein Gels. Transfers were performed on Bio-Rad Trans-Blot Turbo Transfer System using Mini PVDF Transfer Packs. Blocking was performed with LI-COR Odyssey blocking buffer, and antibody staining was performed using blocking buffer with 0.5% Triton-X. KIF26A blotting was performed using mouse (Sigma SAB1407288) and rabbit (Novus NBP2-14158) anti-KIF26A antibody at 1:500 dilution for 2 hours at room temperature, followed by IRDye 800CW goat anti-mouse or anti-rabbit secondary antibody at 1:3000 dilution for 1 hour at room temperature. Blotting for mCherry was performed using chicken anti-mCherry antibody at 1:2000 dilution for 2 hours at room temperature, followed by IRDye 800CW donkey anti-chicken at 1:3000 dilution for 1 hour at room temperature. Images were captured using LI-COR Odyssey Infrared Imaging System. The intensities of the blot bands were measured using the Gel Tool function in ImageJ. The intensity of KIF26A bands were normalized to mCherry to account for variability in transfection efficiency. 4 separately cultured wells of cells were analyzed for each condition with two different antibodies for KIF26A (2 wells for each antibody).

#### Microtubule stability assays in SH-SY5Y cells

SH-SY5Y cells were grown as adherent cultures to 30% confluency. Empty mCherry plasmid, mCherry-tagged WT human KIF26A and KIF26A patient variants expression plasmids were transfected with Lipofectatmine 3000 reagent. For microtubule depolymerization, cells 48 hr post-transfection were treated with 10μM Nocodazole for 15 min and fixed, followed by immunostaining for βIII-tubulin and Acetylated tubulin. Random areas containing 2-5 mCherry^+^ cells were imaged using Zeiss Observer Z1 inverted fluorescent microscope at 63X magnification. The same image acquisition parameters were used for the comparison between cells transfected with different plasmids. Cells with depolymerized tubulin displayed cloudy βIII-tubulin signal dispersed in the cytoplasm instead of the normal fibrous morphology. The number of GFP+ cells with and without microtubule depolymerization for each condition were counted across 15 randomly imaged areas. To evaluate changes in microtubule stability with KIF26A KD, GFP-tagged Scramble shRNA, hKIF26A shRNA1, hKIF26A shRNA2 were transfected with Lipofectatmine 3000 reagent. The cells were checked for GFP reporter expression at 24 hr and fixed 48 hr post-transfection by 4% PFA in PBS for 15 minutes, followed by immunostaining for acetylated tubulin and detyrosinated tubulin. Random areas containing 2-5 mCherry+ cells were imaged using Zeiss Observer Z1 inverted fluorescent microscope at 63X magnification. The average fluorescence intensity for acetylated tubulin and detyrosinated tubulin were measured blindly in ImageJ software.

#### N2A cell culture, transfection and qPCR validation of shRNA KD efficiency

N2A cells were grown as adherent cultures to 60% confluency. Scramble shRNA, mKif26a shRNA1, mKif26a shRNA2 and mKif26a shRNA3 plasmids were transfected onto the N2A cultures with Lipofectamine LTX reagent according to manufacturer protocol. The cells were checked under fluorescence for mCherry reporter expression to estimate transfection efficiency at 24 hr and 48 hr. The transfection efficiency was approximately 70% when the cells were harvested 48 hr post-transfection using a Qiagen RNEasy RNA extraction kit. mRNAs were isolated from the RNA extracts through SuperScript VILO cDNA synthesis using poly-A priming, and qPCR was performed on diluted cDNA using PowerUP SYBR Green Master Mix. Each plasmid was transfected onto three separate replicates, and each transfection replicate was then run with 3 technical replicates in qPCR to estimate the amount of *Kif26a* transcript in the Scramble vs. mKif26a shRNA conditions.

#### KIF26A variant effect prediction using MUpro

To predict the effect of patient missense variants on the protein stability of KIF26A, the cloud computing tool MUpro was used (http://mupro.proteomics.ics.uci.edu/). MUpro is a set of machine learning programs to predict how single-site amino acid mutation affects protein stability. The Support Vector Machines (SVM) and Neural Networks (NN) were trained on a large mutation dataset and show accuracy above 84% via 20-fold cross validation. On the web-based tool, the mutation name, mutation position, original amino acid, substitute amino acid, sequence of WT human KIF26A was imputed. The Prediction results using SVM and NN were respectively summarized for the 5 patient missense variants in Figure S2, with the corresponding Delta Delta G values and confidence scores.

#### mRNA *in situ* hybridization

mRNA *in situ* hybridization was performed on human fetal cortex sections and brain organoid sections using the RNAscope^™^ Multiplex Fluorescent V2 Assay. Custom RNAscope^™^ probe for human *KIF26A* was designed and made by ACD and was 20zz pairs targeting region 2001-3074 of NM_015656.1, which targets both *KIF26A* transcript variants listed on NCBI. The manufacturer’s standard protocol for multiplex fluorescent *in situ* hybridization using HybEZ^™^ Hybridization System was followed. Whole tissue mRNA *in situ* hybridization imaging was performed on a Zeiss LSM 700 confocal microscope with image tiling and stitching, Z- stack of 10μm, and 2x averaging at 20X magnification.

#### Mouse *in utero* electroporation

*In utero* electroporation (IUE) were performed as previously described (Borrell et al., 2005; Saito, 2006; Szczurkowska et al., 2016; Zhang et al., 2016) on pregnant mouse dams at gestational day 13.5. Briefly, the ventricles of individual embryos were injected with approximately 2μL of a 1μg/μL mixture of plasmid DNA using a pulled glass micropipette. 50-volt electric pulses (5 x 50ms pulses at 1.1 second intervals) were then delivered to the brain using paddle electrodes oriented over the dorsal area of the cranium. All procedures were approved under a Boston Children’s Hospital Institutional Animal Care and Use Committee (IACUC)-approved protocol.

#### Mouse tissue preparation

Following 2-day, 4-day or 10-day survival period after the IUE, all mice were euthanized and embryonic brains (for 2-day and 4-day) and postnatal brains (for 10-day) were removed on ice and immersed in 4% paraformaldehyde and 30% sucrose solution overnight. Sucrose-protected brains were then embedded in OCT, stored at −80°C and later sectioned at 30μm using a cryostat.

#### Tissue immunohistochemistry and microscopy

Cryosection slides for different sample types were prepared with some differences for immunohistochemistry. Antigen retrieval was performed only for primary human fetal cortex slides and mouse brain sections by heating slides to 75°C in an oven for 1 hr in Retrievagen A solution, followed by PBS wash twice. The samples were permeabilized with 0.5% Triton-X in PBS for 1 hr (30 min for neurospheres in chamber slides) and blocked with blocking solution of 10% donkey serum in PBS and 0.05% Triton-X for 30 min. Primary antibodies diluted 1:500 in blocking solution were applied to the sections overnight at 4 °C. The primary antibodies used are summarized in the Key Resource Table. After washing with PBST for a minimum of 5 times, secondary antibodies and DAPI (1:2000) diluted in blocking solution were applied to the sections for 1-4 hrs at room temperature or overnight at 4 °C. Secondary anti-bodies were: AlexaFluor 488, 555, 594, or 647 -conjugated donkey antibodies (Invitrogen) and DyLight Fluor 488, 550, and 650 -conjugated donkey antibodies (ThermoFisher) used at 1:500 dilution. Finally, sections were washed with PBST for a minimum of 5 times before mounting with Vectashield Vibrance Antifade Mounting Medium. Images were captured by a Zeiss LSM 700 or Zeiss LSM 980 confocal microscope. Z-stack function was used to image a 10-20 μm thickness unless otherwise specified, and tile-stitching function was used when necessary. Sample images were prepared in ImageJ and Photoshop software.

#### Mouse IUE neuronal migration, neuronal morphology and cell death analysis

For neuronal migration analysis, mouse brain slides were immunostained with primary antibodies for mCherry, TBR2(for cortex IUE), SATB2 (for cortex IUE) and Gad67 (for GE IUE). Images of electroporated region were taken with a Zeiss LSM 700 or Zeiss LSM 980 confocal microscope using 20X objective with tiling/stitching and Z-stack enabled. The basal (pial) and apical (ventricular) surfaces, and the position coordinates of mCherry^+^ electroporated cells were manually marked using the “Cell Counter” plugin in ImageJ in a blinded manner. A custom python script normalized the cells’ laminar positions relative to the total distance from apical to basal surfaces. The frequency distributions of the relative laminar positions in 10 evenly divided bins were calculated and plotted in GraphPad Prism software. The same confocal images were used for morphological analysis between bipolar and unipolar neurons after electroporation. Bipolar and unipolar mCherry^+^ SATB2^+^ neurons were counted manually in ImageJ in a blinded manner, and normalized to the total number of mCherry^+^ SATB2^+^ neurons in each image to calculate the proportionality.

Cell death in electroporated mouse cortex was analyzed by indirect TUNEL staining on tissue cryosections using the ApopTag Fluorescein *in situ* Apoptosis Detection Kit following the manufacturer’s instructions. Electroporated areas were imaged using Zeiss LSM 700 or Zeiss LSM 980 confocal microscope without z-stack. The size of electroporated area was measured and the number of fluorescein^+^ dead cells was counted in ImageJ software in a blinded manner and statistically analyzed in Excel.

#### HiPSC-derived neuron neurite outgrowth assay

Human iPSCs were differentiated into dorsal forebrain excitatory neurons using STEMdiff^™^ SMADi Neural Induction Kit and STEMdiff^™^ Forebrain Neuron Differentiation Kit. The “monolayer protocol” from the manufacturer’s manual was followed. Briefly, iPSCs at around 90% confluency were detached with Accutase and plated on Matrigel-coated 6-well plate at 2 million cells per well. From Day 0 to Day 13, the neural progenitor cells (NPC) were maintained in STEMdiff^™^ Neural Induction Medium + SMADi passaged weekly at 1 million cells per well. At Day 18, the NPCs were passaged into STEMdiff^™^ Forebrain Neuron Differentiation Medium and maintained for an addition of 7 days. Neuron precursors at Day 25 were harvested with Accutase and re-plated into Poly-D-lysine coated glass bottom 12-well plates at 7x 10^4 cells per well, and cultured in BrainPhys^™^ Neuronal Medium and SM1 Kit, supplemented with 1mg/mL mouse laminin and 1X pen/strep.

The day neurons were re-plated were counted as Day 0, and neurons were cultured to Day 5 and Day 21 before fixation and analysis with immunostaining. Neurons were fixed in 4% PFA in PBS for 15 minutes. The cells were permeabilized with 0.5% Triton-X in PBS for 30 min and blocked with blocking solution of 10% donkey serum in PBS and 0.05% Triton-X for 15 min. Primary antibodies for DCX, TUJ1 and NeuN were diluted (1:500) in blocking solution and applied to the sections overnight at 4 °C. After washing with PBST for a minimum of 3 times, secondary antibodies and DAPI (1:2000) diluted in blocking solution were applied to the sections for 1 hour at room temperature. Secondary anti-bodies were: AlexaFluor 488, 555, 594, or 647 -conjugated donkey antibodies (Invitrogen) used at 1:500 dilution. Finally, cells were washed with PBS for a minimum of 3 times before automated imaging with ImageXpress Micro High-Content Imaging System using widefield 20X magnification. Then center of each well (area of approximately 3.2mm x 2.4mm) were automatically imaged as an 8 x 8 grid tiling. Each well was treated as a biological replicate. Total TUJ1^+^ neurite length and number of NeuN^+^ nuclei in each field of view were automatically and unbiasedly quantified using a MetaXpress custom neurite application module. The average neurite length was calculated by dividing total neurite length with NeuN^+^ nuclei number.

#### HiPSC-derived neurosphere migration assay

For differentiation of neurospheres, iPSCs at around 90% confluency were detached with ReLeSR and dissociated with a plate vortex on Day 0. Cells were collected in mTeSR plus medium supplemented with 10μM ROCK inhibitor Y-27632, and seeded into 24-well AggreWell microwell culture plate at 1.5 million cells per well to make embryoid bodies (EBs) of 5,000 cells, followed by centrifuging at 100g for 3 minutes. On Day 1, EBs were transferred from AggreWell to ultra-low attachment 6 well plate with a wide-bore P1000 pipette, and cultured on an orbital shaker placed in the incubator at 100 rotations per minute (rpm) to prevent EBs from aggregating. mTeSR plus medium and EB Medium (AggreWell EB formation medium supplemented with 2 μM Dorsomorphine, 2 μM A83-01 and 1X Penicillin/Streptomycin) were mixed 1:1 for Day 1. On Day 2 and Day 4, full medium change was performed with EB Medium. On Day 5 and Day 6, half of the medium was replaced with Induction Medium consisting of DMEM:F12, 1X N2 Supplement, 1X Penicillin/Streptomycin, 1X Non-essential Amino Acids, 1X GlutaMax, 1 μM CHIR99021, and 1 μM SB-431542. Medium was changed every two days to Day 20. On Day 20, about 50 neurospheres were transferred to one Matrigel-coated chamber slides (Southern Labware) and placed stationary to allow neurospheres to adhere to the surface. The neurospheres were spread on the chamber slide to ensure sufficient distance separating neurospheres and their migratory areas. Two days later (Day 20+2), the neurospheres on chamber slides were fixed with 4% PFA in PBS for 15 minutes at room temperature, washed 3 times with PBS and ready for immunohistochemistry followed by imaging. For the rescue experiment, iPSCs were infected with lentivirus expressing mCherry-CMV>hKIF26A on the passage before EB formation (Day −7).

For comparison of the neuroblast migration distances between control and KIF26A KO neurospheres, random neurospheres were imaged using Zeiss Observer Z1 inverted fluorescent microscope. The entire area including all the neuronal processes and migrating cells around a neurosphere was imaged and tile stitching was used when necessary. The distances of DCX^+^, Pax6^−^ cells migrating away from the bulk of the neurosphere were then manually measured in ImageJ software in a blinded manner, and statistically analyzed in Excel and GraphPad Prism. The frequency distribution of distances was calculated for each individual neurosphere before being averaged for graphical representation.

#### Generation of forebrain organoids

Generation of forebrain organoids from iPSCs was performed as previously described (Qian et al., 2018; Qian et al., 2016a), but with several modifications to adapt the protocol to feeder-free iPSC cultures. On Day 0, iPSCs at around 90% confluency were detached with ReLeSR and dissociated with a plate vortex. Cells were collected in mTeSR plus medium supplemented with 10μM ROCK inhibitor Y-27632, and seeded into 24-well AggreWell microwell culture plate at 3 million cells per well to make embryoid bodies (EBs) of 10,000 cells, followed by centrifuging at 100g for 3 minutes. On Day 1, EBs were transferred from AggreWell to ultra-low attachment 6 well plate with a wide-bore P1000 pipette, and cultured on an orbital shaker placed in the incubator at 100 rpm to prevent EBs from aggregating. mTeSR plus medium and EB Medium (AggreWell EB formation medium supplemented with 2 μM Dorsomorphine, 2 μM A83-01 and 1X Penicillin/Streptomycin) were mixed 1:1 for Day 1. On Day 2 and Day 4, full medium change was performed with EB Medium. On Day 5 and Day 6, half of the medium was replaced with induction medium consisting of DMEM:F12, 1X N2 Supplement, 1X Penicillin/Streptomycin, 1X Non-essential Amino Acids, 1X GlutaMax, 1 μM CHIR99021, and 1 μM SB-431542. On Day 7, organoids were embedded in Matrigel and cultured in the induction medium for 7 more days without orbital shaker. On Day 14, embedded organoids were mechanically dissociated from Matrigel by manual pipetting in a 5 ml pipette tip. Typically, 20 organoids were transferred to each well of an ultra-low attachment 6-well plate containing Differentiation Medium consisting of DMEM:F12, 1X N2 and B27 Supplements, 1X Penicillin/Streptomycin, 1X 2-Mercaptoenthanol, 1X Non-essential Amino Acids, 2.5 μg/ml human insulin, placed on an orbital shaker at 100 rpm. From Day 42 to Day 70, extracellular matrix (ECM) proteins were supplemented in differentiation medium by dissolving Matrigel at 1% (v/v). At Day 70, Differentiation Medium was replaced by Maturation Medium consisting of Neurobasal medium, 1X B27 Supplement, 1X Penicillin/Streptomycin, 1X 2-Mercaptoenthanol, 0.2 mM Ascorbic Acid, and 1 μM cAMP.

#### Organoid tissue preparation

Whole organoids were fixed in 4% PFA in PBS for 30 mins at room temperature. Organoids were washed 3 times with PBS and then immersed in 30% sucrose solution overnight at 4 °C. Organoids were embedded in tissue freezing medium and sectioned with a cryostat (Leica) at 30 μm thickness unless otherwise specified.

#### Analyses of organoid layer thickness, cell death, progenitor proliferation and cleavage angle

Forebrain organoids from Day 50 to Day 100 have well-defined cytoarchitecture that can be distinguished into the VZ, SVZ and CP with immunostaining for CTIP2, TBR2 and SOX2. The VZ was defined by exclusive SOX2 immunoreactivity and neural tube-like morphology at the ventricular (apical) surface. The SVZ was defined by the region containing mixed population of SOX2^+^, CTIP2^+^ and TBR2^+^ nuclei outside the VZ. The CP was defined by the region from the boundary of the SVZ to the outer (basal) surface containing exclusively CTIP2^+^ nuclei. The relative layer thickness analysis was performed similarly to previously described method (Qian et al., 2016a). Random cortical structures were imaged under a confocal microscope and 15 μm z-stacks were projected with maximum intensity in ImageJ software. After defining the boundaries of each layer, the layer thickness was measured in ImageJ in a blinded manner. For each cortical structure, three measurements were taken at 45° angle pointing towards the basal surface and taken average. The relative layer thickness for each layer is calculated by normalizing layer thickness to the sum of VZ, SVZ and CP thicknesses.

For quantification of apoptotic cell death, forebrain organoids were immunostained with Cleaved-Caspase-3 (Cas3), CTIP2 and SOX2. Random cortical structures were imaged under a Zeiss LSM700 confocal microscope at without z-stack. The VZ, SVZ and CP were determined by CTIP2 and SOX2 staining. The number of Cas3^+^ cells and total DAPI-stained nuclei were counted separately for VZ, SVZ and CP in ImageJ software in a blinded manner and statistically analyzed in Excel.

For quantification of cell proliferation, forebrain organoids were immunostained for PH3, SOX2 and TBR2. Random cortical structures were imaged using a Zeiss LSM700 confocal microscope at single z-plane. The number of PH3^+^ SOX2^+^ TBR2^−^, and PH3^+^, TBR2^+^ dividing cells and total DAPI-stained nuclei in the VZ were manually counted in ImageJ software in a blinded manner. The same samples were imaged at 40X under the confocal microscope with a 20μm z-stack thickness at the ventricular surface for measurement of ventricular radial glia cleavage angle. After maximum intensity projection of the z-stack, some PH3^+^ cell pairs show clear cleavage axis, the angel between cleavage axis and the apical surface was measured manually in ImageJ software.

#### EdU labeling and neuronal migration analysis in organoid

Forebrain organoids at Day 53 and Day 70 were pulsed with 1 μM EdU for 1 hr. The medium was then replaced and organoids were washed 3 times with PBS to remove residual EdU. After 8 days, organoids were fixed for immunohistochemistry and EdU detection using Click-iT^®^ EdU Alexa Fluor^®^ 488 Imaging Kit according to the manufacturer’s manual. Cortical structures were randomly imaged under a confocal microscope at the single z-plane. For neuronal migration analysis, the positions of CTIP2^+^ and TBR2^−^ EdU^+^ nuclei were manually marked using the “Cell Counter” plugin in ImageJ in a blinded manner. Their y-coordinates on the image were recorded and normalized to the total distance from apical to basal surfaces of cortical structures to measure their relative laminar positions. The frequency distributions of the relative vertical positions in 10 evenly divided bins of CTIP2^+^ and TBR2^−^ EdU+ nuclei were calculated and plotted in GraphPad Prism software. IPC localization analysis was performed similarly, but only CTIP2^−^ and TBR2^+^ EdU^+^ nuclei were manually marked. For the rescue experiments, DMSO or 0.5μM of FAK inhibitor GSK2256098 was added to culture medium at Day 53 after EdU exposure for 8 days.

#### Human gene expression analysis

The Allen Human Brain Atlas (ABA) publishes a rich dataset of cortical genetic expression across cortical brain regions, from age 8 weeks post conception to adult ages(Miller et al., 2014). BrainSpan data analysis of *KIF26A* was performed with the BrainSpan web browser (https://www.brainspan.org/rnaseq/search/index). RNA-seq expression measured in RPKM (reads per kilobase exon per million mapped reads) was obtained from the BrainSpan project data and summarized to Gencode v10 exons for all annotated neocortical tissues aged 8 weeks post conception to 40 years. “All cortical areas” for Figure 2A include: dorsolateral prefrontal cortex; ventrolateral prefrontal cortex; anterior (rostral) cingulate (medial prefrontal) cortex; orbital frontal cortex; primary motor-sensory cortex; parietal neocortex; primary somatosensory cortex; posteroventral (inferior) parietal cortex; primary auditory cortex; temporal neocortex; posterior (caudal) superior temporal cortex (area 22c), inferolateral temporal cortex (area 20); occipital neocortex and primary visual cortex.

#### Single cell RNA-seq data generation, processing and quality control

Libraries of single cells from dissociated organoids (loaded at a concentration of approximately 1000cells/μL) were prepared using the Chromium Next GEM Single Cell 3' Reagent Kit v3.1 protocol from 10x Genomics. Sequencing was performed on a NovaSeq 6000 (BPF Genomics Core Facility, Harvard Medical School), and transcriptome alignment on Cellranger software (version 4.0.0 version 5.0.0), with reference GRCh38-1.2.0. Cellranger-output filtered feature matrices for each organoid sample were loaded into R using Seurat function Read10X.

Seurat objects were constructed for each organoid. Cells were filtered if they contained less than 200 features. Features were filtered if they were expressed in less than 3 cells. Percent mitochondrial and percent ribosomal transcripts were calculated for each cell as high percent mitochondrial and/or ribosomal transcripts can indicate low-quality cells. Cells with >5% mitochondrial transcripts and/or >20% ribosomal transcripts were filtered. In addition, cells with an unusually high number of features (>= 90th percentile) or unusually low number of features (<= 10th percentile) were filtered, as these are likely doublets and dying cells, respectively. Normalization and variance-stabilization of each Seurat object was performed using Seurat function *SCTransform*. In addition to regression of percent mitochondrial transcripts and percent ribosomal transcripts, the difference between Seurat’s assigned G2M and S phase scores was regressed for each cell. This removes the effect of cell cycle differences between cycling progenitor cells while retaining the differences in cell cycle phase between non-cycling and cycling cells. All cell cycle scores were assigned using the Seurat function *CellCycleScoring.*

#### Integration of organoid scRNA-seq data

Individual Seurat objects for each organoid sample were integrated using Seurat’s *PrepSCTIntegration, FindIntegrationAnchors, and IntegrateData* functions as well as Seurat’s reference-based and reciprocal-PCA workflows. These workflows were picked due to their efficient and accurate performance on large scRNA-seq datasets. Briefly, *PrepSCTIntegration* ensures that all normalized counts have been calculated using *SCTransform* and are present within each Seurat object. *FindIntegrationAnchors* finds anchors, or cross-dataset pairs of cells that are of the same biological state, in a reduced-dimension reciprocal PCA space. We designated sample M_90_G12 as a reference sample due to its technical high-quality, reducing the number of computations required to integrate our 15 organoid samples. Finally, *IntegrateData* creates an integrated Seurat object containing all cells from our 15 organoid samples by leveraging the cross-dataset anchors identified previously. Genes shared between all organoid samples (n = 15,058) were used as features for integration.

#### Cell type annotation and visualization of gene expression patterns for organoid scRNA-seq data

To facilitate visualization and annotation of our integrated Seurat object, a low-dimensionality embedding was produced using Seurat function *RunPCA*. An elbow plot revealed that approximately 13 principal components (PCs) captured the majority of the variation and a UMAP was constructed using the first 13 PCs with Seurat function *RunUMAP*. Finally, unsupervised clusters were calculated using Seurat functions *FindNeighbors* and *FindClusters* (resolution = 0.25). One cluster (cluster 10) was identified to primarily consist of low-quality cells due to low number of features and transcripts per cell and was subsequently removed from downstream analysis. *RunPCA, RunUMAP, FindNeighbors, and FindClusters*(resolution = 0.25) was repeated to yield the final UMAP embedding and unsupervised cluster assignments.

Unsupervised clusters were annotated via the examination of the expression of established neurodevelopmental marker genes using Seurat function *FeaturePlot*. Ultimately, 9 cell types were assigned: Immature excitatory neurons (ImmatEx), mature excitatory neurons (MatEx), migration excitatory neurons (MigEx), ventricular radial glia (vRG), intermediate progenitor cells (IPC), outer radial glia (oRG), inhibitory neurons (Inhib), dividing progenitor cells 1 (DivPg1), and dividing progenitor cells 2 (DivPg2).

#### Processing and integration of published scRNAseq datasets to build fetal human cortex atlas

Raw count matrices and relevant metadata were obtained from relevant repositories for

1. Polioudakis et al., *Neuron* 2019 (http://solo.bmap.ucla.edu/) (Polioudakis et al., 2019)
2. Fan et al., *Cell Res.* 2018 (GEO: GSE103723) (Fan et al., 2018)
3. Nowakowski et al., *Science* 2017 (https://cells.ucsc.edu/?ds=cortex-dev) (Nowakowski et al., 2017)
4. Zhong et al., *Nature* 2018 (GEO: GSE104276). (Zhong et al., 2018)

Seurat objects were constructed for each dataset using the Seurat function

##### CreateSeuratObject

Cells were filtered if they contained less than 200 features. Features were filtered if they were expressed in less than 3 cells. Each dataset’s provided metadata, including cell type annotation, was used without modification. Percent mitochondrial were calculated for each cell as high percent mitochondrial can indicate low-quality cells. Cells with >5% mitochondrial transcripts were filtered. In addition, cells with an unusually high number of features (>= 90^th^ percentile) or unusually low number of features (<= 10^th^ percentile) were filtered, as these are likely doublets and dying cells, respectively. Normalization and variance-stabilization of each Seurat object was performed using Seurat function *SCTransform.* In addition to regression of percent mitochondrial transcripts and percent ribosomal transcripts, the difference between Seurat’s assigned G2M and S phase scores was regressed for each cell. This removes the effect of cell cycle differences between cycling progenitor cells while retaining the differences in cell cycle phase between non-cycling and cycling cells. All cell cycle scores were assigned using the Seurat function *CellCycleScoring.* Finally, the following dataset-specific covariates were also regressed:

1. *Polioudakis et al.,:* library, index, gestation_week
2. *Fan et al.,:* brain_region, batch, sex, gestation_week
3. *Nowakowski et al.,:* name, age_in_weeks
4. *Zhong et al.,:* pfc, week

One dataset, Polioudakis et al., showed significant clustering by tissue donor. As recommended by Seurat developers, we addressed this source of variation by integrating across donors using Seurat functions *FindIntegrationAnchors* and *IntegrateData. FindIntegrationAnchors* finds anchors, or cross-dataset pairs of cells that are of the same biological state, in a reduced-dimension reciprocal PCA space. *IntegrateData* creates an integrated Seurat object containing all cells all donors by leveraging the cross-dataset anchors identified previously. All genes in the Polioudakis dataset were used as features for anchors.

To facilitate consistent cell-type annotations across cells from each individual dataset, we used Seurat’s label transfer analysis pipeline to transfer cell-type labels from the Polioudakis et al., dataset to cells from the other datasets (queries). To do so, we used the Seurat functions *FindTransferAnchors* and *TransferData*. *FindTransferAnchors* finds anchors, or cross-dataset pairs of cells, by projecting a query dataset onto a reference dataset. TransferData uses the identified transfer anchors to assign each query cell a predicted cell type based on the reference dataset’s annotations. Overall, we saw high concordance among the transferred cell labels, the query datasets’ original cell labels, and cell labels defined based on marker gene expression.

We integrated each of our dataset-specific Seurat objects using Seurat functions *FindIntegrationAnchors* and *IntegrateData*. *FindIntegrationAnchors* finds anchors, or cross-dataset pairs of cells that are of the same biological state in a reduced dimension CCA space. *IntegrateData* creates an integrated Seurat object containing all cells all donors by leveraging the cross-dataset anchors identified previously. 3000 integration features were identified using the Seurat function *FindIntegrationFeatures*, which identifies highly variable genes across multiple datasets. To facilitate visualization of our integrated Seurat object, a low-dimensionality embedding was produced using Seurat function *RunPCA*. An elbow plot revealed that 15 principal components (PCs) captured the majority of the variation and a tSNE was constructed using the first 15 PCs with Seurat function *RunTSNE*. Labels transferred from the Polioudakis et al., dataset (explained above) were used as cell-type annotations with cells of the same type clustering together preferentially in low-dimensional space, as expected.

#### Comparison of fetal atlas and organoid scRNA-seq data

To facilitate consistent cell-type annotations across cells from each individual dataset, we used Seurat’s label transfer analysis pipeline to transfer cell-type labels from the integrated fetal atlas to our integrated organoid Seurat object. To do so, we used the Seurat functions *FindTransferAnchors* and *TransferData. FindTransferAnchors* finds anchors, or cross-dataset pairs of cells, by projecting a query dataset onto a reference dataset. *TransferData* uses the identified transfer anchors to assign each query cell a predicted cell type based on the reference dataset’s annotations. Overall, we saw high concordance among the transferred cell labels and the cell labels defined based on unsupervised clustering and marker gene expression.

To calculate similarity between cells of the same type in the fetal atlas versus integrated organoid Seurat object, we determined the gene-specific average expression for each cell type within our organoid and fetal atlas datasets. We then cross-correlated each cell type in the organoid and fetal atlas datasets, revealing specific and high correlation between cells of the same type in the organoid and fetal atlas datasets.

#### Trajectory inference and pseudotime analysis for organoid scRNA-seq data

Trajectory inference and pseudotime analysis was performed using R package monocle3. We created a cell_data_set object from our integrated Seurat object using monocle3 function *as.cell_data_set.* We then learned a low-dimensionality trajectory using monocle3 functions *cluster_cells, learn_graph,* and *order_cells.* As monocle3 requires identification of root cells, we randomly sampled 100 annotated DivPg1 cells as our root cells. Pseudotime values for each cell were visualized with Seurat’s *FeaturePlot* function after being calculated with monocle3. Visualization of gene expression through pseudotime was done using custom scripts and ggplot2 to create a smoothed heatmap of expression vs pseudotime.

#### Differential expression analysis, gene ontology analysis and gene set enrichment analysis for organoid scRNA-seq data

Differentially expressed genes between WT and KO organoid samples were identified for each annotated cell type using Seurat function *FindMarkers*. MAST was used to perform differential expression testing, due to its superior performance over other scRNA-seq differential expression tools in benchmarking studies. Since the differential expression results were used to perform gene set enrichment analysis (GSEA, described in following section) and to create volcano plots, we set the *FindMarkers* parameters *logfc.threshold* and min.pct to 0. This allowed us to determine the average log-fold change and associated p-value for every gene without filtering, as is required for GSEA and the volcano plot visualization. We called a gene as differentially expressed if it had an ∣log-fold change∣ > 0.25 and an adjusted p-value < 0.05.

Volcano plots were created using the R package *EnhancedVolcano’s EnhancedVolcano* function using the differential expression results calculated using *FindMarkers*. Venn diagrams of differentially expressed genes between different clusters were created with R package *ggvenn’s ggvenn* function. For gene ontology analysis, differentially expressed genes between KO and WT organoids in the Migrating Excitatory Neuron (MigEx) clusters were selected at a cut of adjusted p value < 0.05, and Log_2_FC > ±1. Functional enrichment with gene ontology was implemented with DAIVD Functional Annotation Tool (DAVID Bioinformatics Resources 6.8, NIAID/NIH), setting Benjamini-Hochberg FDR < 0.05. Gene set enrichment analysis (GSEA) was performed for each cell type based on genes identified as differentially expressed between cells of that type in WT vs KO organoids. To perform GSEA, the limma function *geneSetTest* was used with default parameters. Adjusted p-values were calculated with Bonferroni adjustment using the R package stat’s function *p.adjust.* Visualization of GSEA results was performed with custom scripts and clusterProfiler’s *gseaplot2* function.

### QUANTIFICATION AND STATISTICAL ANALYSIS

Individual organoids are treated as biological replicates, unless otherwise indicated in the Figure Legends. Data are presented as mean ± S.E.M. or mean + S.D., unless otherwise indicated in the Figure Legends. Statistical analyses were performed using the student’s t-test in Excel or Prism software. Significance was defined by P-value < 0.05. Organoid samples were randomly taken from the culture for experiments and analysis. Sample sizes are determined empirically. The sample sizes are designed to account for the variability between organoids and human iPSC cell lines and match current standards in human brain organoid-related studies. Other statistical details of experiments can be found in the Figure Legends. No data were excluded.

## Supplementary Material

Supplementary figures and figure legend

## Figures and Tables

**Table:** KEY RESOURCES TABLE

REAGENT or RESOURCE	SOURCE	IDENTIFIER
**Primary Antibodies**		
Cleaved Caspase-3 (Asp175)	Cell Signaling	9661
CTIP2	Abcam	ab18465
DCX	Santa Cruz	sc-8066
GAD-65/67	Santa Cruz	sc-365180
GFP	Aves	GFP-1020
KI67	BD	550609
KIF26A	Sigma	HPA046882-100UL
KIF26A	Sigma	SAB1407288-50UG
KIF26A	Novus	NBP2-14158-25ul
mCherry	Abcam	ab205402
Nestin	Aves	NES
NeuN	Millipore	MAB377
Neuropilin-1	R&D Systems	AF3870-SP
PAX6	BD	561664
Phospho-FAK (Tyr576)	ThermoFisher	700013
Phospho-Histone H3 (Ser10)	Cell Signaling	9706S
SATB2	Abcam	ab51502
SOX2	Santa Cruz	sc-17320
SOX2	R&D Systems	AF2018
TBR2	Sigma	HPA028896-100UL
Acetylated tubulin	Sigma	T7451-100UL
βIII-tubulin	Abcam	ab18207
Detyrosinated tubulin	Sigma	AB3201
**Chemicals, Peptides, and Recombinant Proteins**		
DMEM:F12	Invitrogen	cat. # 11330032
Neurobasal medium	Gibco	cat. # 21103049
Phosphate Buffered Saline	Gibco	cat. # 10010023
Non-essential Amino Acids	Gibco	cat. # 11140050
Penicillin/Streptomycin	Gibco	cat. # 15140122
2-Mercaptoenthanol	Gibco	cat. # 21985023
Glutamax	Gibco	cat. # 35050061
Dorsomorphine	StemCell Technologies	cat. # 72102
A83-01	StemCell Technologies	cat. # 72022
N2 Supplement	Gibco	cat. # 17502048
B27 Supplements	Gibco	cat. # 17504044
CHIR99021	StemCell Technologies	cat. # 72052
SB-431542	StemCell Technologies	cat. # 72232
Y-27632	StemCell Technologies	cat. # 72304
Nocodazole	StemCell Technologies	cat. # 74072
GSK2256098	Selleckchem	cat. # S8523
Matrigel	Corning	cat. # 354230
mTeSR^™^ Plus	StemCell Technologies	cat. # 100-0276
ReLeSR	StemCell Technologies	cat. # 05872
STEMdiff^™^ SMADi Neural Induction Kit	StemCell Technologies	cat. # 08582
STEMdiff^™^ Forebrain Neuron Differentiation Kit	StemCell Technologies	cat. # 08600
BrainPhys^™^ Neuronal Medium and SM1 Kit	StemCell Technologies	cat. # 05792
Human Insulin solution	Sigma	cat. # I0516
Ascorbic Acid	Sigma	cat. # 1043003
Cyclic AMP	Sigma	cat. # A9501
Paraformaldehyde (PFA)	Polysciences	cat. # 18814-10
Sucrose	Sigma	cat. # S5016
Tissue freezing medium	General Data	cat. # TFM-5
Triton-X	Sigma	cat. # T9284
donkey serum	Millipore	cat. # S30
VECTASHIELD Vibrance Antifade Mounting Medium	Vector Laboratories	cat. # H-1700-10
Retrievagen Antigen Retrieval Systems	Fisher	cat. # BDB550524
RIPA Lysis and Extraction Buffer	ThermoFisher	cat. # 89901
Anti-Adherence Rinsing Solution	StemCell Technologies	cat. # 07010
Lipofectamine 3000	ThermoFisher	cat. # L3000008
Lipofectamine LTX	ThermoFisher	cat. # 15338030
Poly-D-Lysine	ThermoFisher	cat. # A3890401
Laminin Mouse Protein, Natural	ThermoFisher	cat. # 23017015
Fast Green FCF	Sigma	cat. # F7252
Precast Protein Gels	Bio-Rad	cat. # 4561093
Mini PVDF Transfer Packs	Bio-Rad	cat. # 1704156EDU
**Critical Commercial Assays**		
RNAscope^™^ Multiplex Fluorescent V2 Assay	ACD	cat. # 323110
RNAscope^®^ Probe- Hs-KIF26A-C1	ACD	cat. # 856751
RNAscope^®^ Probe- Hs-RBFOX3-C3	ACD	cat. # 415591-C3
RNAscope^®^ Probe- Hs-RORB-C2	ACD	cat. # 446061-C2
Click-iT^®^ EdU Alexa Fluor^®^ 488 Imaging kit	ThermoFisher	cat. # C10337
ApopTag^®^ Fluorescein In Situ Apoptosis Detection Kit	Millipore	cat. # S7110
NovaSeq 6000 S1 Reagent Kit v1.5 (100 cycles)	Illumina	cat. # 20028319
Chromium Next GEM Single Cell 3' Reagent Kit v3.1	10x Genomics	PN-1000121, PN-1000127
**Recombinant DNA and Viral Vectors**		
Lentivirus pLV[Exp]-mCherry-CMV>hKIF26A	VectorBuilder	VB200617-1157zbr
DNA plasmid pLV[Exp]-mCherry-CMV>hKIF26A[NM_015656.1]	VectorBuilder	VB200617-1157zbr
DNA plasmid pLV[Exp]-mCherry-CMV>{hKIF26A[NM_015656.1]*(R721C)}	VectorBuilder	VB210408-1104wqh
DNA plasmid pLV[Exp]-mCherry-CMV>{hKIF26A[NM_015656.1]*(P949S)}	VectorBuilder	VB210408-1092ysc
DNA plasmid pLV[Exp]-mCherry-CMV>{hKIF26A[NM_015656.1]*(A1559V)}	VectorBuilder	VB210408-1106ccf
DNA plasmid pLV[Exp]-mCherry-CMV>{hKIF26A[NM_015656.1]*R1624C}	VectorBuilder	VB220131-1120adp
DNA plasmid pRP[shRNA]-mCherry-U6>mKif26a[shRNA#1]	VectorBuilder	VB200226-1310hxy
DNA plasmid pRP[shRNA]-mCherry-U6>mKif26a[shRNA#2]	VectorBuilder	VB200226-1311htj
DNA plasmid pRP[shRNA]-mCherry-U6>mKif26a[shRNA#3]	VectorBuilder	VB200226-1312eag
DNA plasmid pLV[shRNA]-EGFP-U6>Scramble_shRNA#1	VectorBuilder	VB010000-0001mty
DNA plasmid pLV[msgRNA]-mCherry-U6>Scramble[msgRNA#1]	VectorBuilder	VB200617-1158rnm
DNA plasmid pLV[shRNA]-EGFP-U6>hKIF26A[shRNA#1]	VectorBuilder	VB010000-0001mty
DNA plasmid pLV[shRNA]-EGFP-U6>hKIF26A[shRNA#2]	VectorBuilder	VB200817-1831kku
**Deposited Data**		
Single cell RNA sequencing data of organoids	This paper	Accession code: XXX
**Experimental Models: Cell Lines**		
Neuro-2a (N2A) mouse neuroblast cell line	ATCC	CCL-131
HEK293T human kidney epithelial cell line	ATCC	CRL-3216
SH-SY5Y human neuroblastoma epithelial cell line	ATCC	CRL-2266
Human WT iPSC line “PGP1” derived from healthy male fibroblasts	Synthego (Lee et al., 2009)	PGP1
Isogenic human iPSC line derived from “PGP1”, with frameshift mutation to knockout KIF26A gene using CRISPR-Cas9 editing	This paper	KO D17
Isogenic human iPSC line derived from “PGP1”, with frameshift mutation to knockout KIF26A gene using CRISPR-Cas9 editing	This paper	KO N19
Human WT iPSC line “280” derived from healthy male fibroblasts	Boston Children’s Hospital hESC Core (Schlaeger et al., 2015)	280
Isogenic human iPSC line derived from “280”, with frameshift mutation to knockout KIF26A gene using CRISPR-Cas9 editing	This paper	KO D2
Isogenic human iPSC line derived from “280”, with frameshift mutation to knockout KIF26A gene using CRISPR-Cas9 editing	This paper	KO G4
**Experimental Models: Organisms/Strains**		
CD1 Mice	Charles River	Crl:CD1(ICR)
Ferrets	Marshall Bioresources	N/A
**Software and Algorithms**		
Zeiss Zen (Blue edition)	Zeiss	https://www.zeiss.com/microscopy/us/products/microscope-software/zen.html
NextSeq System Suite v2.0	Illumina	https://support.illumina.com/downloads/nextseq-system-suite-v2-0.html
ImageJ (Fiji)	NIH	https://imagej.nih.gov/ij/docs/guide/146-2.html
GraphPad Prism (8.0)	GraphPad Software Inc., La Jolla, CA, USA	https://www.graphpad.com/scientific-software/prism/
Microsoft Excel	Microsoft	https://www.microsoft.com/en-us/p/excel/cfq7ttc0k7dx?activetab=pivot%3aoverviewtab
Adobe Photoshop (CC)	Adobe	https://www.adobe.com/products/photoshop.html
Adobe Illustrator (CC)	Adobe	https://www.adobe.com/products/illustrator.html
10x Genomics Cell Ranger 4.0.0 and 5.0.0	10x Genomics	https://support.10xgenomics.com/single-cell-gene-expression/software/pipelines/latest/release-notes
TopHat (2.0)	Johns Hopkins University	https://ccb.jhu.edu/software/tophat/index.shtml
R, R studio with R package Seurat (v 2.3.4)	RStudio	https://cran.r-project.org/web/packages/Seurat/index.html
R studio with edgeR (v2.15) package	RStudio	https://www.ncbi.nlm.nih.gov/pmc/articles/PMC4023662/
R package ClustifyR	RStudio	https://github.com/rnabioco/clustifyr
MathWorks Matlab	MathWorks	https://www.mathworks.com/
MetaXpress	Molecular Devices	https://www.moleculardevices.com/products/cellular-imaging-systems/acquisition-and-analysis-software/metaxpress#gref
**Other**		
AggreWell^™^400	StemCell Technologies	cat. # 34425
12 Well glass bottom plate with high performance #1.5 cover glass	Cellvis	cat. # P12-1.5H-N
Costar^®^ 6-well Ultra-Low Attachment Plate	Corning	cat. # 3471

## References

[R1] An L, Li W, Hu X, Zhang W, and Zhao S (2018). Abundant Focal Adhesion Kinase Causes Aberrant Neuronal Migration Via Its Phosphorylation at Tyr925. J Mol Neurosci 64, 102–110 DOI: 10.1007/s12031-017-1010-1. 29209901

[R2] Asselin L, Rivera Alvarez J, Heide S, Bonnet CS, Tilly P, Vitet H, Weber C, Bacino CA, Baranano K, Chassevent A, (2020). Mutations in the KIF21B kinesin gene cause neurodevelopmental disorders through imbalanced canonical motor activity. Nat Commun 11, 2441 DOI: 10.1038/s41467-020-16294-6. 32415109 PMC7229210

[R3] Bahi-Buisson N, Poirier K, Fourniol F, Saillour Y, Valence S, Lebrun N, Hully M, Bianco CF, Boddaert N, Elie C, (2014). The wide spectrum of tubulinopathies: what are the key features for the diagnosis? Brain 137, 1676–1700 DOI: 10.1093/brain/awu082. 24860126

[R4] Bershteyn M, Nowakowski TJ, Pollen AA, Di Lullo E, Nene A, Wynshaw-Boris A, and Kriegstein AR (2017). Human iPSC-Derived Cerebral Organoids Model Cellular Features of Lissencephaly and Reveal Prolonged Mitosis of Outer Radial Glia. Cell Stem Cell 20, 435–449 e434 DOI: 10.1016/j.stem.2016.12.007. 28111201 PMC5667944

[R5] Besset V, Scott RP, and Ibanez CF (2000). Signaling complexes and protein-protein interactions involved in the activation of the Ras and phosphatidylinositol 3-kinase pathways by the c-Ret receptor tyrosine kinase. J Biol Chem 275, 39159–39166 DOI: 10.1074/jbc.M006908200. 10995764

[R6] Borrell V, Yoshimura Y, and Callaway EM (2005). Targeted gene delivery to telencephalic inhibitory neurons by directional in utero electroporation. J Neurosci Methods 143, 151–158 DOI: 10.1016/j.jneumeth.2004.09.027. 15814147

[R7] Calalb MB, Polte TR, and Hanks SK (1995). Tyrosine phosphorylation of focal adhesion kinase at sites in the catalytic domain regulates kinase activity: a role for Src family kinases. Mol Cell Biol 15, 954–963 DOI: 10.1128/MCB.15.2.954. 7529876 PMC231984

[R8] Chaussepied M, and Ginsberg D (2004). Transcriptional regulation of AKT activation by E2F. Mol Cell 16, 831–837 DOI: 10.1016/j.molcel.2004.11.003. 15574337

[R9] Cheng J, Randall A, and Baldi P (2006). Prediction of protein stability changes for single-site mutations using support vector machines. Proteins 62, 1125–1132 DOI: 10.1002/prot.20810. 16372356

[R10] Edwards TJ, Sherr EH, Barkovich AJ, and Richards LJ (2014). Clinical, genetic and imaging findings identify new causes for corpus callosum development syndromes. Brain 137, 1579–1613 DOI: 10.1093/brain/awt358. 24477430 PMC4032094

[R11] Emenecker RJ, Griffith D, and Holehouse AS (2021). Metapredict: a fast, accurate, and easy-to-use predictor of consensus disorder and structure. Biophys J 120, 4312–4319 DOI: 10.1016/j.bpj.2021.08.039. 34480923 PMC8553642

[R12] Fan X, Dong J, Zhong S, Wei Y, Wu Q, Yan L, Yong J, Sun L, Wang X, Zhao Y, (2018). Spatial transcriptomic survey of human embryonic cerebral cortex by single-cell RNA-seq analysis. Cell Res 28, 730–745 DOI: 10.1038/s41422-018-0053-3. 29867213 PMC6028726

[R13] Geng A, Qiu R, Murai K, Liu J, Wu X, Zhang H, Farhoodi H, Duong N, Jiang M, Yee JK, (2018). KIF20A/MKLP2 regulates the division modes of neural progenitor cells during cortical development. Nat Commun 9, 2707 DOI: 10.1038/s41467-018-05152-1. 30006548 PMC6045631

[R14] Girskis KM, Stergachis AB, DeGennaro EM, Doan RN, Qian X, Johnson MB, Wang PP, Sejourne GM, Nagy MA, Pollina EA, (2021). Rewiring of human neurodevelopmental gene regulatory programs by human accelerated regions. Neuron DOI: 10.1016/j.neuron.2021.08.005. PMC8542612 34478631

[R15] Hirokawa N, Niwa S, and Tanaka Y (2010). Molecular motors in neurons: transport mechanisms and roles in brain function, development, and disease. Neuron 68, 610–638 DOI: 10.1016/j.neuron.2010.09.039. 21092854

[R16] Janisch KM, Vock VM, Fleming MS, Shrestha A, Grimsley-Myers CM, Rasoul BA, Neale SA, Cupp TD, Kinchen JM, Liem KF Jr., (2013). The vertebrate-specific Kinesin-6, Kif20b, is required for normal cytokinesis of polarized cortical stem cells and cerebral cortex size. Development 140, 4672–4682 DOI: 10.1242/dev.093286. 24173802 PMC3833427

[R17] Jiang SX, Sheldrick M, Desbois A, Slinn J, and Hou ST (2007). Neuropilin-1 is a direct target of the transcription factor E2F1 during cerebral ischemia-induced neuronal death in vivo. Mol Cell Biol 27, 1696–1705 DOI: 10.1128/MCB.01760-06. 17178835 PMC1820462

[R18] Jumper J, Evans R, Pritzel A, Green T, Figurnov M, Ronneberger O, Tunyasuvunakool K, Bates R, Zidek A, Potapenko A, (2021). Highly accurate protein structure prediction with AlphaFold. Nature 596, 583–589 DOI: 10.1038/s41586-021-03819-2. 34265844 PMC8371605

[R19] Konjikusic MJ, Gray RS, and Wallingford JB (2021). The developmental biology of kinesins. Dev Biol 469, 26–36 DOI: 10.1016/j.ydbio.2020.09.009. 32961118 PMC10916746

[R20] Lancaster MA, and Knoblich JA (2014). Organogenesis in a dish: modeling development and disease using organoid technologies. Science (New York, NY) 345, 1247125 DOI: 10.1126/science.1247125 [doi]. 25035496

[R21] Lawrence CJ, Dawe RK, Christie KR, Cleveland DW, Dawson SC, Endow SA, Goldstein LS, Goodson HV, Hirokawa N, Howard J, (2004). A standardized kinesin nomenclature. J Cell Biol 167, 19–22 DOI: 10.1083/jcb.200408113. 15479732 PMC2041940

[R22] Lee JH, Park IH, Gao Y, Li JB, Li Z, Daley GQ, Zhang K, and Church GM (2009). A robust approach to identifying tissue-specific gene expression regulatory variants using personalized human induced pluripotent stem cells. PLoS Genet 5, e1000718 DOI: 10.1371/journal.pgen.1000718. 19911041 PMC2766639

[R23] Liberzon A, Birger C, Thorvaldsdottir H, Ghandi M, Mesirov JP, and Tamayo P (2015). The Molecular Signatures Database (MSigDB) hallmark gene set collection. Cell Syst 1, 417–425 DOI: 10.1016/j.cels.2015.12.004. 26771021 PMC4707969

[R24] Little JN, and Dwyer ND (2019). p53 deletion rescues lethal microcephaly in a mouse model with neural stem cell abscission defects. Hum Mol Genet 28, 434–447 DOI: 10.1093/hmg/ddy350. 30304535 PMC6337704

[R25] Ma RR, Zhang H, Chen HF, Zhang GH, Tian YR, and Gao P (2021). MiR-19a/miR-96-mediated low expression of KIF26A suppresses metastasis by regulating FAK pathway in gastric cancer. Oncogene 40, 2524–2538 DOI: 10.1038/s41388-020-01610-7. 33674746

[R26] Midorikawa R, Takei Y, and Hirokawa N (2006). KIF4 motor regulates activity-dependent neuronal survival by suppressing PARP-1 enzymatic activity. Cell 125, 371–383 DOI: 10.1016/j.cell.2006.02.039. 16630823

[R27] Miki H, Okada Y, and Hirokawa N (2005). Analysis of the kinesin superfamily: insights into structure and function. Trends Cell Biol 15, 467–476 DOI: 10.1016/j.tcb.2005.07.006. 16084724

[R28] Miller JA, Ding SL, Sunkin SM, Smith KA, Ng L, Szafer A, Ebbert A, Riley ZL, Royall JJ, Aiona K, (2014). Transcriptional landscape of the prenatal human brain. Nature 508, 199–206 DOI: 10.1038/nature13185. 24695229 PMC4105188

[R29] Mitani T, Isikay S, Gezdirici A, Gulec EY, Punetha J, Fatih JM, Herman I, Akay G, Du H, Calame DG, (2021). High prevalence of multilocus pathogenic variation in neurodevelopmental disorders in the Turkish population. Am J Hum Genet 108, 1981–2005 DOI: 10.1016/j.ajhg.2021.08.009. 34582790 PMC8546040

[R30] Moffat JJ, Ka M, Jung EM, and Kim WY (2015). Genes and brain malformations associated with abnormal neuron positioning. Mol Brain 8, 72 DOI: 10.1186/s13041-015-0164-4. 26541977 PMC4635534

[R31] Mukherjee A, Brooks PS, Bernard F, Guichet A, and Conduit PT (2020). Microtubules originate asymmetrically at the somatic golgi and are guided via Kinesin2 to maintain polarity within neurons. Elife 9 DOI: 10.7554/eLife.58943. PMC7394546 32657758

[R32] Niquille M, Garel S, Mann F, Hornung JP, Otsmane B, Chevalley S, Parras C, Guillemot F, Gaspar P, Yanagawa Y, (2009). Transient neuronal populations are required to guide callosal axons: a role for semaphorin 3C. PLoS Biol 7, e1000230 DOI: 10.1371/journal.pbio.1000230. 19859539 PMC2762166

[R33] Niwa S. (2015). Kinesin superfamily proteins and the regulation of microtubule dynamics in morphogenesis. Anat Sci Int 90, 1–6 DOI: 10.1007/s12565-014-0259-5. 25347970

[R34] Noctor SC, Martinez-Cerdeno V, Ivic L, and Kriegstein AR (2004). Cortical neurons arise in symmetric and asymmetric division zones and migrate through specific phases. Nat Neurosci 7, 136–144 DOI: 10.1038/nn1172. 14703572

[R35] Nowakowski TJ, Bhaduri A, Pollen AA, Alvarado B, Mostajo-Radji MA, Di Lullo E, Haeussler M, Sandoval-Espinosa C, Liu SJ, Velmeshev D, (2017). Spatiotemporal gene expression trajectories reveal developmental hierarchies of the human cortex. Science 358, 1318–1323 DOI: 10.1126/science.aap8809. 29217575 PMC5991609

[R36] Pasca SP (2018). The rise of three-dimensional human brain cultures. Nature 553, 437–445 DOI: 10.1038/nature25032. 29364288

[R37] Piper M, Plachez C, Zalucki O, Fothergill T, Goudreau G, Erzurumlu R, Gu C, and Richards LJ (2009). Neuropilin 1-Sema signaling regulates crossing of cingulate pioneering axons during development of the corpus callosum. Cereb Cortex 19 Suppl 1, i11–21 DOI: 10.1093/cercor/bhp027. 19357391 PMC2693530

[R38] Polioudakis D, de la Torre-Ubieta L, Langerman J, Elkins AG, Shi X, Stein JL, Vuong CK, Nichterwitz S, Gevorgian M, Opland CK, (2019). A Single-Cell Transcriptomic Atlas of Human Neocortical Development during Mid-gestation. Neuron 103, 785–801 e788 DOI: 10.1016/j.neuron.2019.06.011. 31303374 PMC6831089

[R39] Pollen AA, Nowakowski TJ, Chen J, Retallack H, Sandoval-Espinosa C, Nicholas CR, Shuga J, Liu SJ, Oldham MC, Diaz A, (2015). Molecular Identity of Human Outer Radial Glia during Cortical Development. Cell 163, 55–67 DOI: 10.1016/j.cell.2015.09.004 [doi]. 26406371 PMC4583716

[R40] Poulton CJ, Schot R, Kia SK, Jones M, Verheijen FW, Venselaar H, de Wit MC, de Graaff E, Bertoli-Avella AM, and Mancini GM (2011). Microcephaly with simplified gyration, epilepsy, and infantile diabetes linked to inappropriate apoptosis of neural progenitors. Am J Hum Genet 89, 265–276 DOI: 10.1016/j.ajhg.2011.07.006. 21835305 PMC3155199

[R41] Qian X, Jacob F, Song MM, Nguyen HN, Song H, and Ming GL (2018). Generation of human brain region-specific organoids using a miniaturized spinning bioreactor. Nat Protoc 13, 565–580 DOI: 10.1038/nprot.2017.152. 29470464 PMC6241211

[R42] Qian X, Nguyen HN, Song MM, Hadiono C, Ogden SC, Hammack C, Yao B, Hamersky GR, Jacob F, Zhong C, (2016a). Brain-Region-Specific Organoids Using Mini-bioreactors for Modeling ZIKV Exposure. Cell 165, 1238–1254 DOI: 10.1016/j.cell.2016.04.032. 27118425 PMC4900885

[R43] Qian X, Nguyen HN, Song MM, Hadiono C, Ogden SC, Hammack C, Yao B, Hamersky GR, Jacob F, Zhong C, (2016b). Brain-Region-Specific Organoids Using Mini-bioreactors for Modeling ZIKV Exposure. Cell DOI: 10.1016/j.cell.2016.04.032. PMC4900885 27118425

[R44] Qian X, Song H, and Ming GL (2019). Brain organoids: advances, applications and challenges. Development 146 DOI: 10.1242/dev.166074. PMC6503989 30992274

[R45] Qian X, Su Y, Adam CD, Deutschmann AU, Pather SR, Goldberg EM, Su K, Li S, Lu L, Jacob F, (2020). Sliced Human Cortical Organoids for Modeling Distinct Cortical Layer Formation. Cell Stem Cell 26, 766–781 e769 DOI: 10.1016/j.stem.2020.02.002. 32142682 PMC7366517

[R46] Rakic P. (2009). Evolution of the neocortex: a perspective from developmental biology. Nat Rev Neurosci 10, 724–735 DOI: 10.1038/nrn2719. 19763105 PMC2913577

[R47] Saito T. (2006). In vivo electroporation in the embryonic mouse central nervous system. Nat Protoc 1, 1552–1558 DOI: 10.1038/nprot.2006.276. 17406448

[R48] Schlaeger TM, Daheron L, Brickler TR, Entwisle S, Chan K, Cianci A, DeVine A, Ettenger A, Fitzgerald K, Godfrey M, (2015). A comparison of non-integrating reprogramming methods. Nat Biotechnol 33, 58–63 DOI: 10.1038/nbt.3070. 25437882 PMC4329913

[R49] Smith RS, Kenny CJ, Ganesh V, Jang A, Borges-Monroy R, Partlow JN, Hill RS, Shin T, Chen AY, Doan RN, (2018). Sodium Channel SCN3A (NaV1.3) Regulation of Human Cerebral Cortical Folding and Oral Motor Development. Neuron 99, 905–913 e907 DOI: 10.1016/j.neuron.2018.07.052. 30146301 PMC6226006

[R50] Sun S, Lei Y, Li Q, Wu Y, Zhang L, Mu PP, Ji GQ, Tang CX, Wang YQ, Gao J, (2017). Neuropilin-1 is a glial cell line-derived neurotrophic factor receptor in glioblastoma. Oncotarget 8, 74019–74035 DOI: 10.18632/oncotarget.18630. 29088765 PMC5650320

[R51] Szczurkowska J, Cwetsch AW, dal Maschio M, Ghezzi D, Ratto GM, and Cancedda L (2016). Targeted in vivo genetic manipulation of the mouse or rat brain by in utero electroporation with a triple-electrode probe. Nat Protoc 11, 399–412 DOI: 10.1038/nprot.2016.014. 26844428

[R52] Tunyasuvunakool K, Adler J, Wu Z, Green T, Zielinski M, Zidek A, Bridgland A, Cowie A, Meyer C, Laydon A, (2021). Highly accurate protein structure prediction for the human proteome. Nature 596, 590–596 DOI: 10.1038/s41586-021-03828-1. 34293799 PMC8387240

[R53] Valiente M, Ciceri G, Rico B, and Marin O (2011). Focal adhesion kinase modulates radial glia-dependent neuronal migration through connexin-26. J Neurosci 31, 11678–11691 DOI: 10.1523/JNEUROSCI.2678-11.2011. 21832197 PMC6623127

[R54] Vasquez RJ, Howell B, Yvon AM, Wadsworth P, and Cassimeris L (1997). Nanomolar concentrations of nocodazole alter microtubule dynamic instability in vivo and in vitro. Mol Biol Cell 8, 973–985 DOI: 10.1091/mbc.8.6.973. 9201709 PMC305707

[R55] Wang J, Shen WH, Jin YJ, Brandt-Rauf PW, and Yin Y (2007a). A molecular link between E2F-1 and the MAPK cascade. J Biol Chem 282, 18521–18531 DOI: 10.1074/jbc.M610538200. 17452331

[R56] Wang L, Dutta SK, Kojima T, Xu X, Khosravi-Far R, Ekker SC, and Mukhopadhyay D (2007b). Neuropilin-1 modulates p53/caspases axis to promote endothelial cell survival. PLoS One 2, e1161 DOI: 10.1371/journal.pone.0001161. 18000534 PMC2048754

[R57] Wang L, Tanaka Y, Wang D, Morikawa M, Zhou R, Homma N, Miyamoto Y, and Hirokawa N (2018). The Atypical Kinesin KIF26A Facilitates Termination of Nociceptive Responses by Sequestering Focal Adhesion Kinase. Cell Rep 24, 2894–2907 DOI: 10.1016/j.celrep.2018.05.075. 30208315

[R58] Xu J, Liu L, Ma R, Wang Y, Chen X, Liu H, Ji Y, Liu T, and Gao P (2020). E2F1 Induces KIF26A Transcription and Promotes Cell Cycle Progression via CDK-RB-E2Fs Feedback Loop in Breast Cancer. Front Oncol 10, 530933 DOI: 10.3389/fonc.2020.530933. 33505901 PMC7832431

[R59] Yokota Y, Gashghaei HT, Han C, Watson H, Campbell KJ, and Anton ES (2007). Radial glial dependent and independent dynamics of interneuronal migration in the developing cerebral cortex. PLoS One 2, e794 DOI: 10.1371/journal.pone.0000794. 17726524 PMC1950908

[R60] Zhang W, and Liu HT (2002). MAPK signal pathways in the regulation of cell proliferation in mammalian cells. Cell Res 12, 9–18 DOI: 10.1038/sj.cr.7290105. 11942415

[R61] Zhang X, Chen MH, Wu X, Kodani A, Fan J, Doan R, Ozawa M, Ma J, Yoshida N, Reiter JF, (2016). Cell-Type-Specific Alternative Splicing Governs Cell Fate in the Developing Cerebral Cortex. Cell 166, 1147–1162 e1115 DOI: 10.1016/j.cell.2016.07.025. 27565344 PMC5248659

[R62] Zhang X, Ling J, Barcia G, Jing L, Wu J, Barry BJ, Mochida GH, Hill RS, Weimer JM, Stein Q, (2014). Mutations in QARS, encoding glutaminyl-tRNA synthetase, cause progressive microcephaly, cerebral-cerebellar atrophy, and intractable seizures. Am J Hum Genet 94, 547–558 DOI: 10.1016/j.ajhg.2014.03.003. 24656866 PMC3980424

[R63] Zhao H, Maruyama T, Hattori Y, Sugo N, Takamatsu H, Kumanogoh A, Shirasaki R, and Yamamoto N (2011). A molecular mechanism that regulates medially oriented axonal growth of upper layer neurons in the developing neocortex. J Comp Neurol 519, 834–848 DOI: 10.1002/cne.22536. 21280039

[R64] Zhong S, Zhang S, Fan X, Wu Q, Yan L, Dong J, Zhang H, Li L, Sun L, Pan N, (2018). A single-cell RNA-seq survey of the developmental landscape of the human prefrontal cortex. Nature 555, 524–528 DOI: 10.1038/nature25980. 29539641

[R65] Zhou R, Niwa S, Guillaud L, Tong Y, and Hirokawa N (2013). A molecular motor, KIF13A, controls anxiety by transporting the serotonin type 1A receptor. Cell Rep 3, 509–519 DOI: 10.1016/j.celrep.2013.01.014. 23438369

[R66] Zhou R, Niwa S, Homma N, Takei Y, and Hirokawa N (2009). KIF26A is an unconventional kinesin and regulates GDNF-Ret signaling in enteric neuronal development. Cell 139, 802–813 DOI: 10.1016/j.cell.2009.10.023. 19914172

[R67] Zhu J, Blenis J, and Yuan J (2008). Activation of PI3K/Akt and MAPK pathways regulates Myc-mediated transcription by phosphorylating and promoting the degradation of Mad1. Proc Natl Acad Sci U S A 105, 6584–6589 DOI: 10.1073/pnas.0802785105. 18451027 PMC2373325

[R68] Zimmermann L, Stephens A, Nam SZ, Rau D, Kubler J, Lozajic M, Gabler F, Soding J, Lupas AN, and Alva V (2018). A Completely Reimplemented MPI Bioinformatics Toolkit with a New HHpred Server at its Core. J Mol Biol 430, 2237–2243 DOI: 10.1016/j.jmb.2017.12.007. 29258817

