# Supplementary material for: Loss of Non-motor Kinesin KIF26A Causes Congenital Brain Malformations via Dysregulated Neuronal Migration and Axonal Growth as well as Apoptosis
Source: Dev Cell. Author manuscript; Available in PMC 2023 Oct 19. (PMC10585591; doi:10.1016/j.devcel.2022.09.011)
Supplement: Supplementary figures and figure legend [file NIHMS1935233-supplement-Supplementary_figures_and_figure_legend.pdf]

Table S1

Identifier	**Individual ID**	**A01**	**B01**	**C01**	**D01**	**E01**
	**Pedigree ID**	BAB10995	PED026C	SHE_1305	PMG14801	BAB5949
Genetics	**Genomic position (hg19)**	14:104642565dupC/14:104642565dupC	14:104640615C>T/14:104643801C>T	14:104643801C>T/14:104643995C>T	14:104641970C>T/ 14:104643801C>T	14:104643929:C>T/14:104643929:C>T
	**CDNA change**	c.3440dupC/c.3440dupC	c.2161C>T/c.4676C>T	c.4676C>T/ c.4870C>T	c.2845C>T/c.4676C>T	c.4804C>T/c.4804C>T
	**Protein change**	p.Ala1148Cysfs*20/p.Ala1148Cysfs*20	p.Arg721Cys/p.Ala1559Val	p.Ala1559Val/ p.Arg1624Cys	p.Pro949Ser/p.Ala1559Val	p.Arg1602Trp/p.Arg1602Trp
	**Transcript**	ENST00000423312/ NM_015656	ENST00000423312/ NM_015656	ENST00000423312/ NM_015656	ENST00000423312/ NM_015656	ENST00000423312/ NM_015656
	**Prior genetic test**	Chromosomal microarray - multiple regions of areas of homozygosity	COL4A1 and COL4A2 Sanger sequencing & chromosomal microarray - normal	None	22q FISH - normal	Karyotype
	**Mode of variant discovery**	Trio WES	Trio WES	Trio WES	Trio WES	Duo ES
Birth History	**Pregnancy history**	n.d.	Significant PV bleeding	Borderline gestational diabetes mellitus	n.d.	
	**Birth history**	PICU due to pneumonia and respiratory insufficiency	Termination of pregnancy	Emergency C-section	SVD requiring forceps	C-section, meconium aspiration
	**Birth gestational age**	n.d.	21 weeks	41 weeks	40 weeks	38 weeks
	**Birth Weight**	n.d.	440g	3.20 kg (-0.58 SD)	3.69kg (.26SD)	2.73 kg (0.1SD)
	**Birth length**	n.d.	26cm	n.d.	50cm (-0.06SD)	45 cm (-0.06SD)
	**Birth head circumference**	n.d.	20.8cm	n.d.	n.d.	n.d.
General	**Biological Sex**	Male	Male	Male	Male	Male
	**Age at last evaluation**	3 month	21 gestational weeks	17 years	30 years	3 years 6 months
Neurologic Abnormalities	**Head Circumference**	36.2 cm (-3.4SD)	n/a	n.d.	55.5cm (0.27SD)	n.d.
	**Neuromuscular**	n.d.	Arthrogryposis	Not present	n.d.	Hypotonia
	**ID/DD**	n.d.	n/a	Learning disability	n.d.	Developmental delay
	**Epilepsy/ Seizures**	n.d.	n/a	Not present	Present	Not present
	**Ventricular abnormality**	Ventriculomegaly	n.d.	Colpocephaly	Hydrocephalus	Mild ventriculomegaly
	**White matter abnormality**	Decreased volume	Poor grey-white differentiation	Not present	n.d.	Not present
	**Corpus Callosum abnormality**	Thin	-3.1SD in length	Agenesis	n.d.	Thin
	**Cortical abnormality**	Cerebral atrophy	Bilateral schizencephaly	Not present	Polymicrogyria	Polymicrogyria
	**Opthalmologic**	Strabismus	n/a	Not present	Divergent left stabismus, bilateral nystagmus	Not present
Systemic Abormalities	**Dysmorphic features**	Narrow palpebral fissure, retrognathia/micrognathia, short neck, short philtrum, bitemporal narrowing, high narrow palate, hypotelorism	Slightly low set left ear, long flat philtrum. high arched palate.	Not present	n.d.	Synophrys, brachydactyly, growth retardation
	**Congential Heart Defects**	n.d.	Not present	Not present	n.d.	Not present
	**GI/GU abnormalities**	Ileus/megacolon	Absent stomach bubble	Not present	n.d.	Normal abdominal ultrasound and blood chemistry

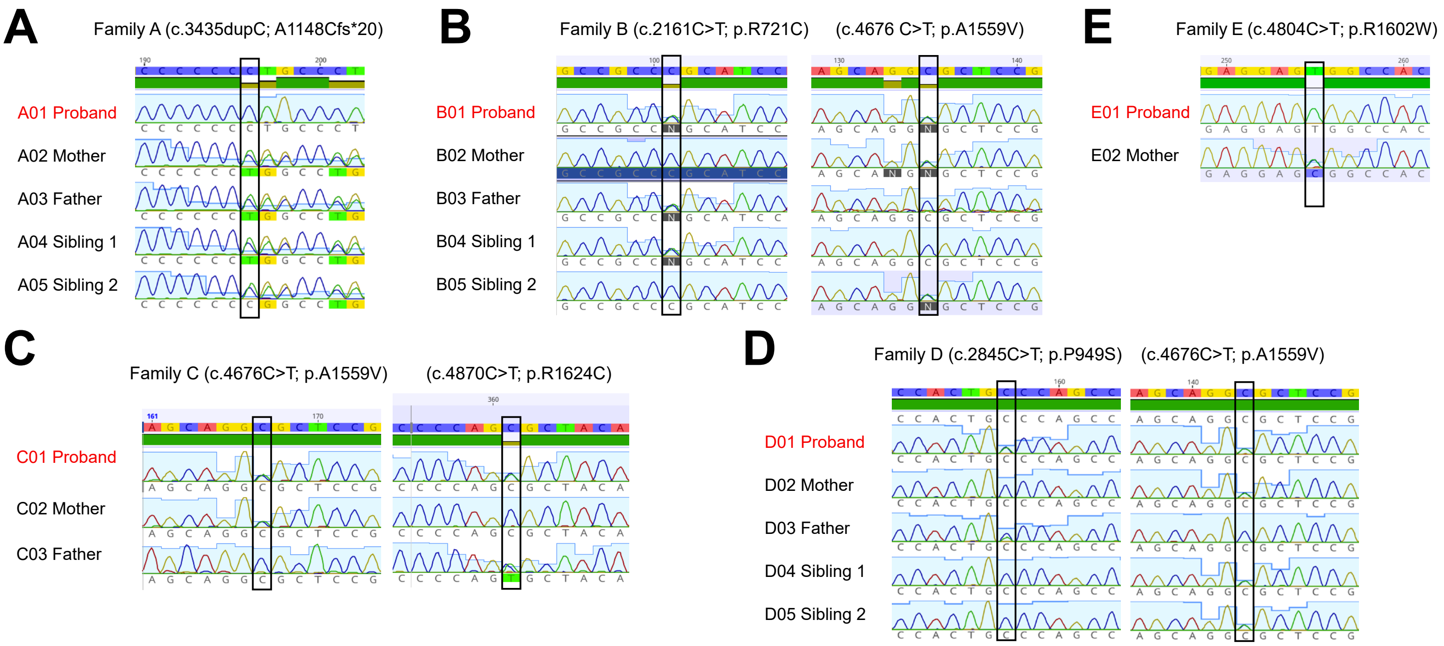

F

Arg721Cys

KIF26A Orthologs AAs 714-728	
Human	STVQLAARIHRLRRK
Chimpanzee	STVQLAARIHRLCRK
Rhesus	STVQLAARIHRLRRK
Mouse	STVQLAARIHRLRRK
Rat	STVQLAARIHRLRRK
Cat	STVQLAARIHRLRRK
Ferret	STVQLAARIHRLRRK
Opossum	TTVQLASRIHRMRKK
Chicken	TTIQLSSRIHRMRKK
Sea Turtle	MTIQLASRIHRMRKK
Xenopus	STVQLASRIHRMKKK
Zebrafish	TTIQLASRIHRMRKK
Lamprey	ATVQLAARVHRLRKK

Ala1559Val

KIF26A Orthologs AAs 1552-1566	
Human	VMGTKQALRAAHSRV
Chimpanzee	IMGTKQALRAAHSRV
Rhesus	IMGTKQALRAAHSRV
Mouse	IMGTKQAFRAAHSRV
Rat	IMGTKQAFRAAHSRV
Cat	IMGTKQALRAAHSRV
Ferret	IMGTRQALRAAHSRV
Opossum	IMGTKQAMRAANSRV
Chicken	IMGTKQAMRAANSRV
Sea Turtle	IMGTKQAIRAANSRV
Xenopus	IMGTKQALRAANNRV
Zebrafish	IMGTKQAMRAANSRV
Lamprey	IMGTKQAIRAAHSRV

Arg1624Cys

KIF26A Orthologs AAs 1617-1631	
Human	TAPRRPQRYSSGHGS
Chimpanzee	TAPRRPQRYSSGHGS
Rhesus	TAPRRPQRYSSGHGS
Mouse	TAPRRPQRYSSGHGS
Rat	TAPRRPQRCSSGHGS
Cat	TAPRRPQRCSSGHGS
Ferret	TAPRRPQRYSSGHGS
Opossum	TAPRRPQRYSSGHGS
Chicken	TAPRRPQRYSSGHGS
Sea Turtle	TAPRRPQRYSSGHGS
Xenopus	TAPRRPQRYSSGHGS
Zebrafish	TAPRRPQRYSSGHGS
Lamprey	TAPRRPHHRSSGHGS

Pro949Ser

KIF26A Orthologs AAs 942-956	
Human	GGRRPLPSPAPPPPQ
Chimpanzee	GGRRPLPSPAPPPPQ
Rhesus	GGRRPLPSPAPPPPQ
Mouse	GGRRPLPSPAPPPPR
Rat	GGRRLLPSPAPPPPR
Cat	GGGRPLPGPAPPPPR
Ferret	GGRRPLPSPAPPPPR
Opossum	-GKRPLPSPAPPPPQ
Chicken	-VKRPLPSPAPPPPQ
Sea Turtle	-VKRPLPSPAPPPPQ
Xenopus	MVRRHLPSPAPPPVQ
Zebrafish	--K--LPKPAPPPPQ
Lamprey	GGRRLLPSPAPPPPR

Arg1602Trp

KIF26A Orthologs AAs 1595-1609	
Human	GVNVGEERPPTGPAL
Chimpanzee	GVNVGEERPPTGPAL
Rhesus	GVNVGEERPPPGPAL
Mouse	GVNLAEERQPSSPAL
Rat	GVNLAEERQPSSPAL
Cat	GVNVGEERPPAGPAL
Ferret	GVNVGEERLPAVPVL
Opossum	GVNLGDEK--PVPAL
Chicken	GINLSDEK--PIPVL
Sea Turtle	GINLSDEK--QIPVL
Xenopus	GINLSDEK--QTPVL
Zebrafish	GVALNEDK--PIPIL
Lamprey	GVQLTDEKPSPSAAL

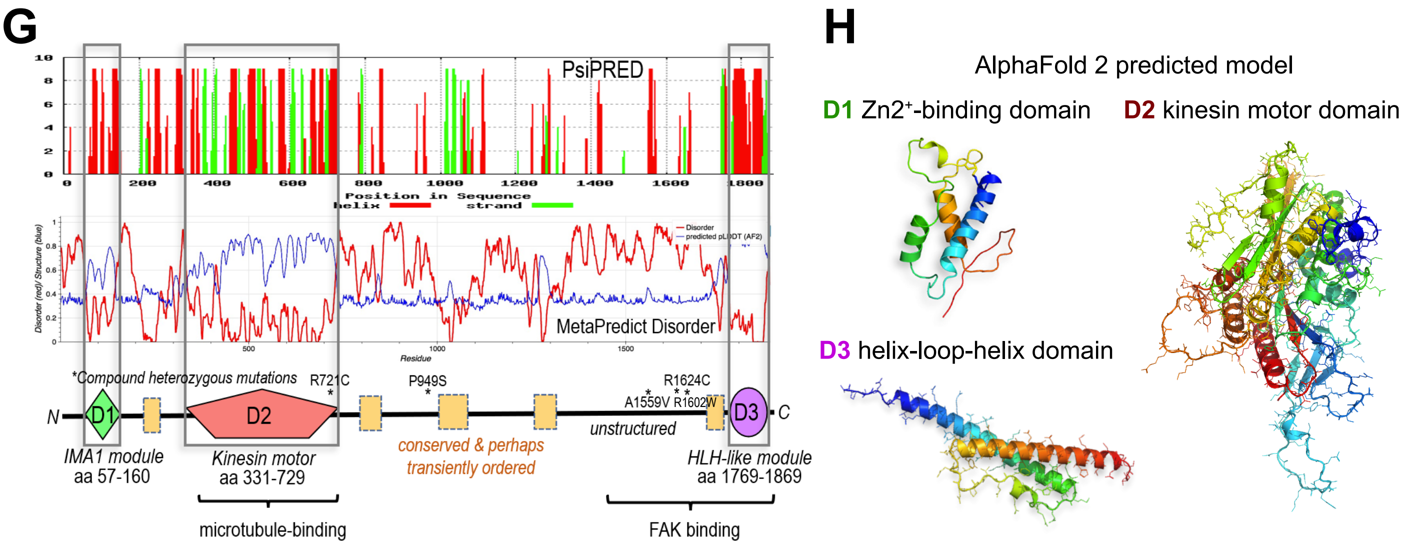

Figure S1 (Qian et al.)

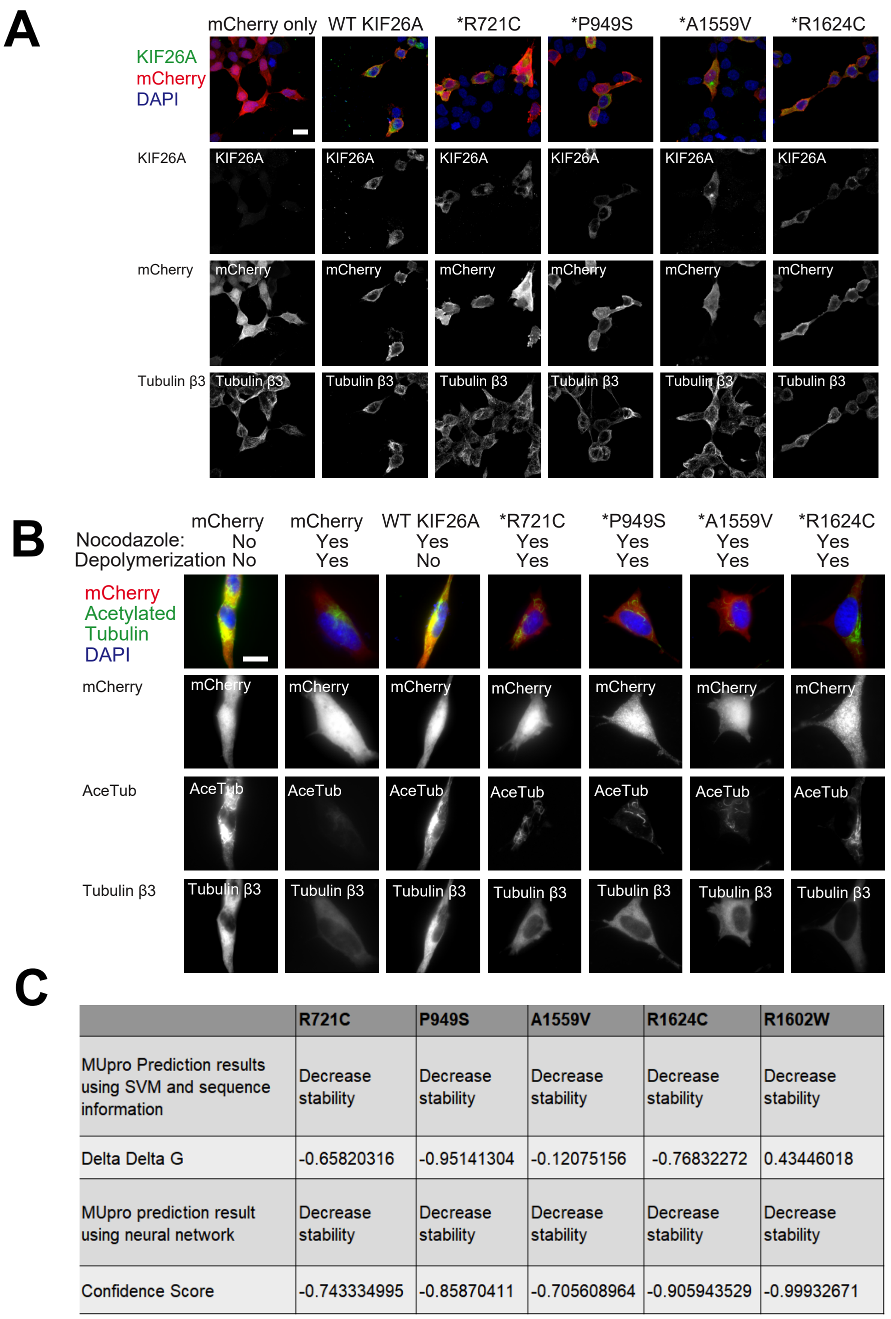

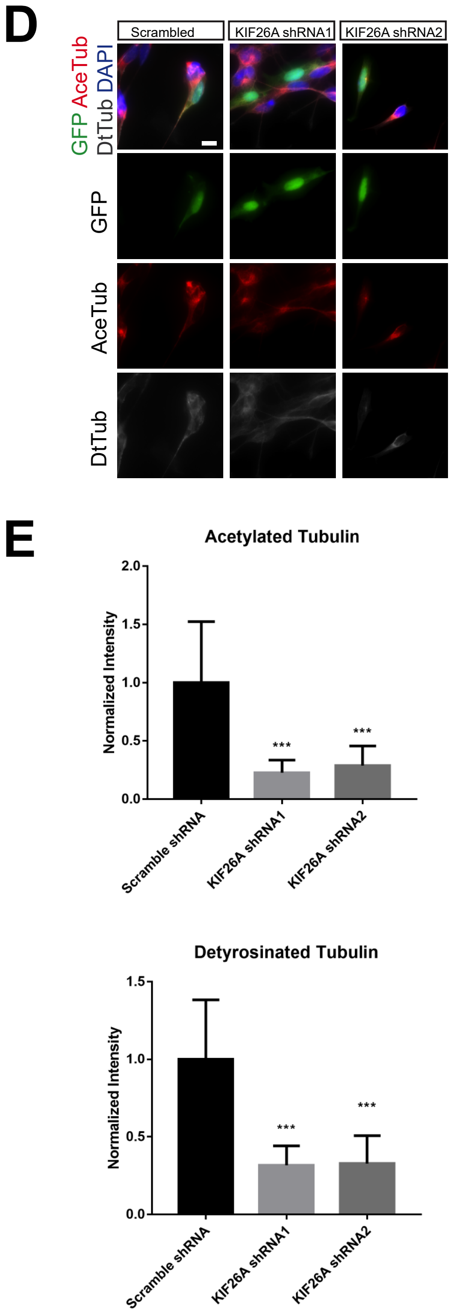

C

	R721C	P949S	A1559V	R1624C	R1602W
MUpro Prediction results using SVM and sequence information	Decrease stability	Decrease stability	Decrease stability	Decrease stability	Decrease stability
Delta Delta G	-0.65820316	-0.95141304	-0.12075156	-0.76832272	0.43446018
MUpro prediction result using neural network	Decrease stability	Decrease stability	Decrease stability	Decrease stability	Decrease stability
Confidence Score	-0.743334995	-0.85870411	-0.705608964	-0.905943529	-0.99932671

Figure S2 (Qian et al.)

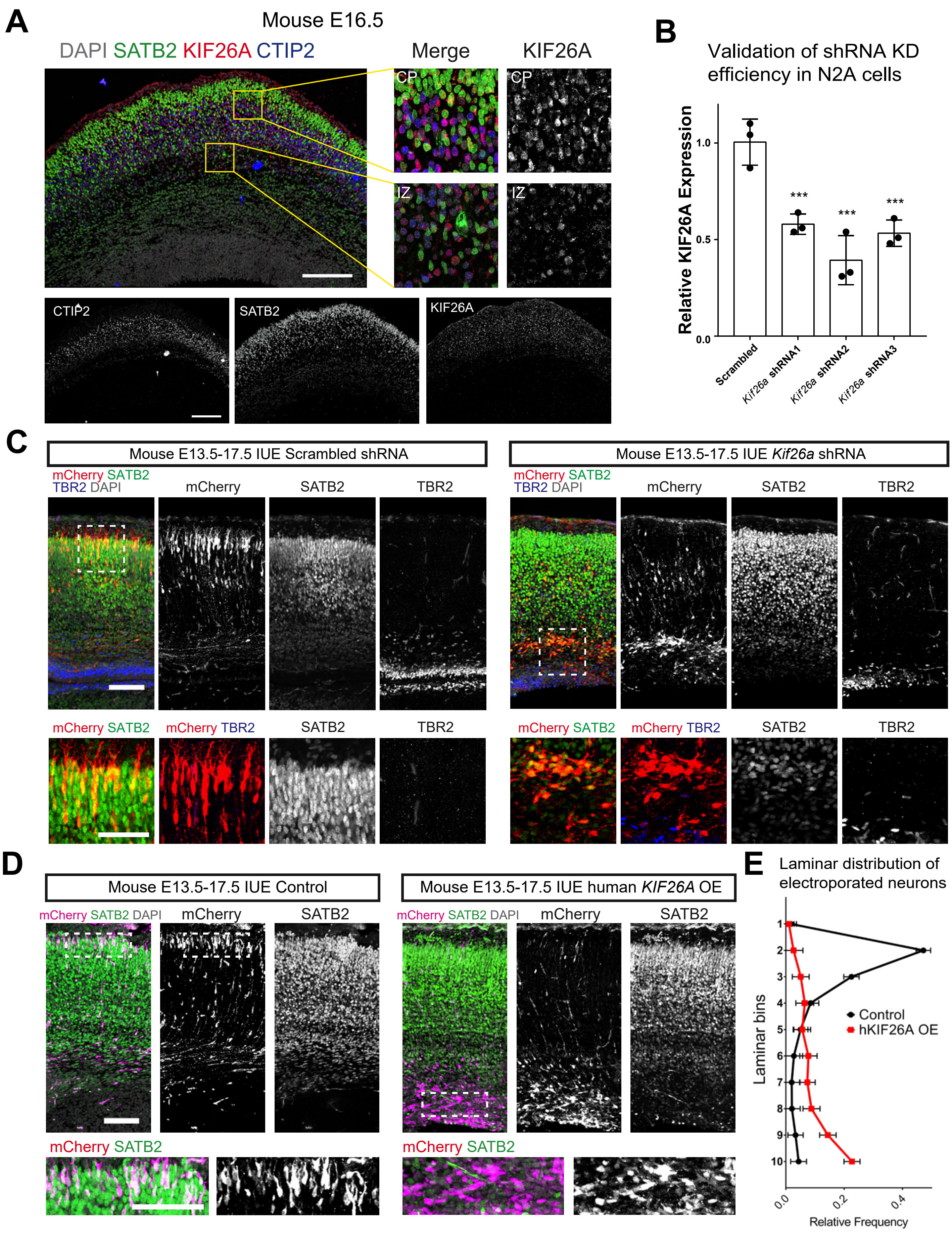

Figure S3 (Qian et al.)

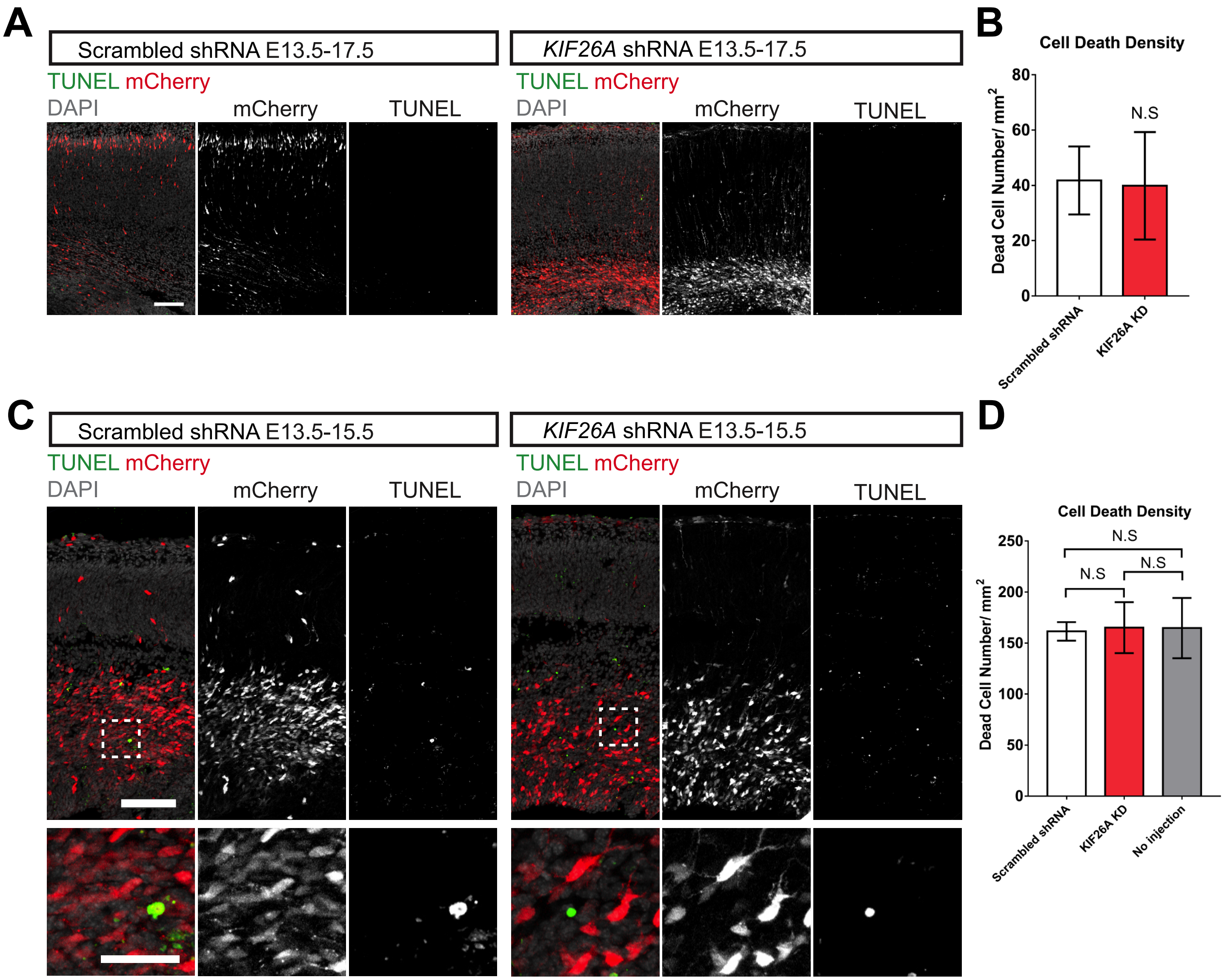

Figure S4 (Qian et al.)

A

KO line name	Parent line	Guide Target	Indel %
D17	PGP1	CGGCCCTGATGGCTTGTCGA	99
N19	PGP1	CGGCCCTGATGGCTTGTCGA	99
D2	280	CCAGCACCACGACCAGCTCG	99
G4	280	CCAGCACCACGACCAGCTCG	99

B

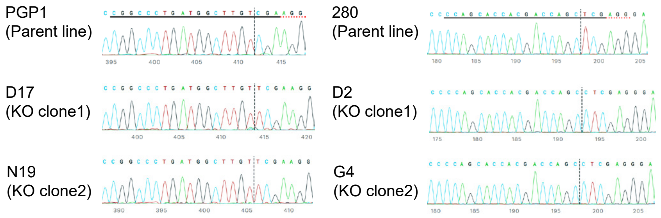

C

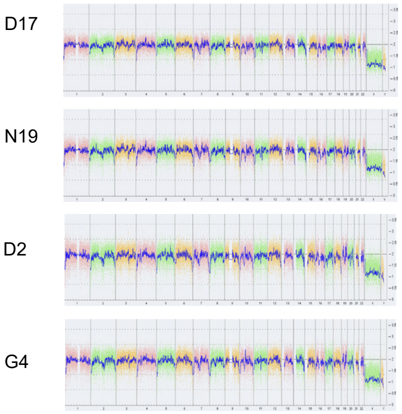

Figure S5 (Qian et al.)

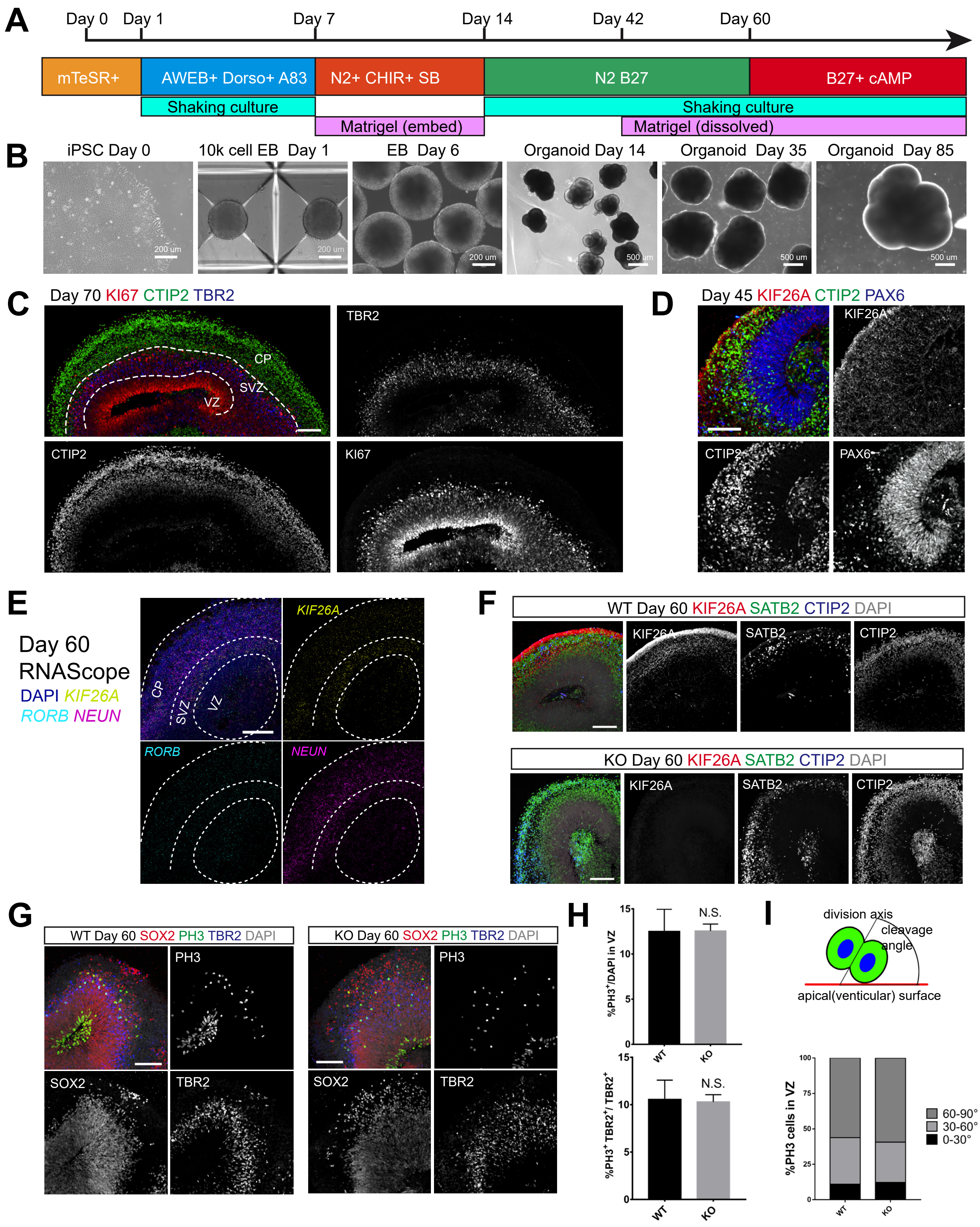

Figure S6 (Qian et al.)

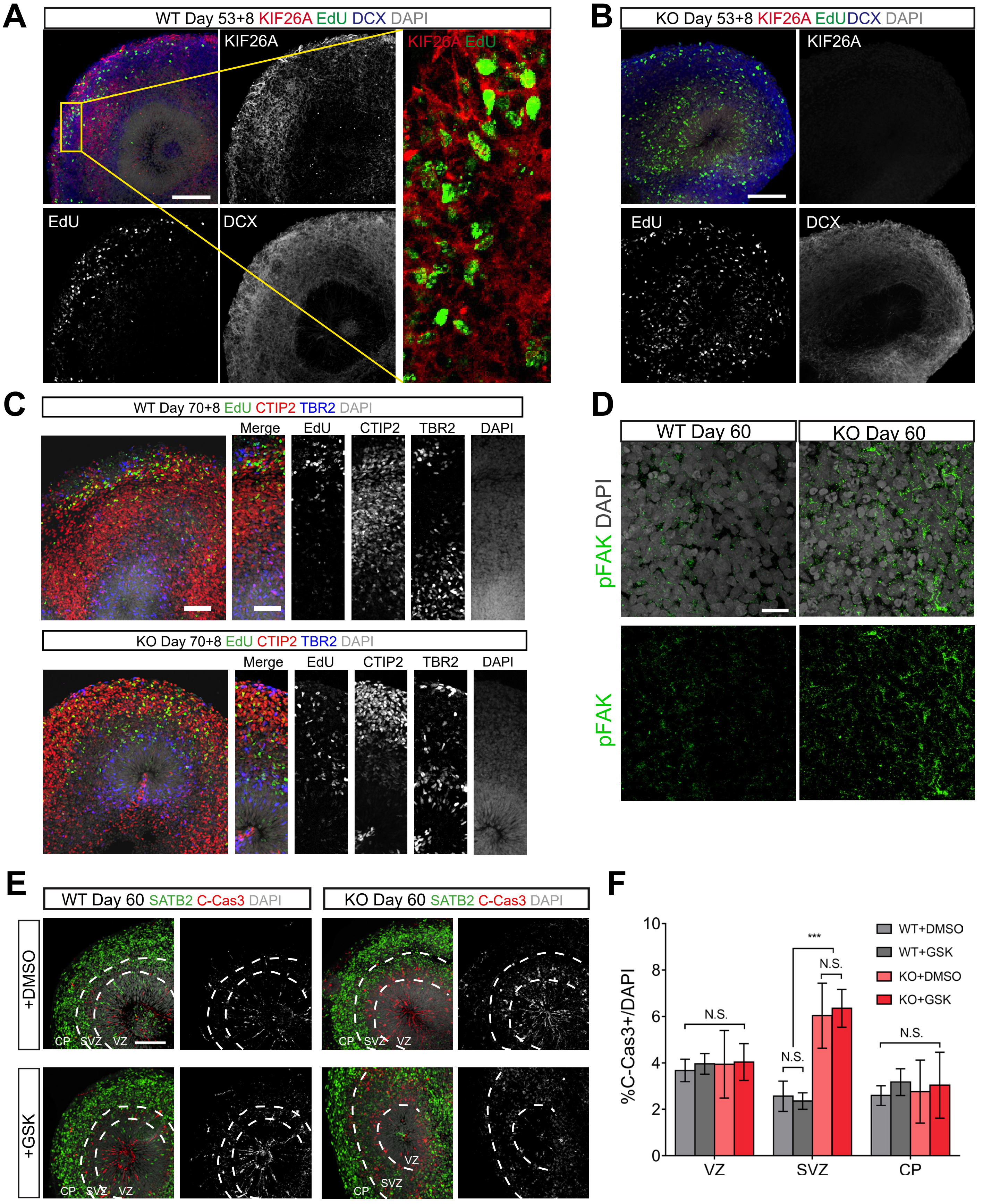

Figure S7 (Qian et al.)

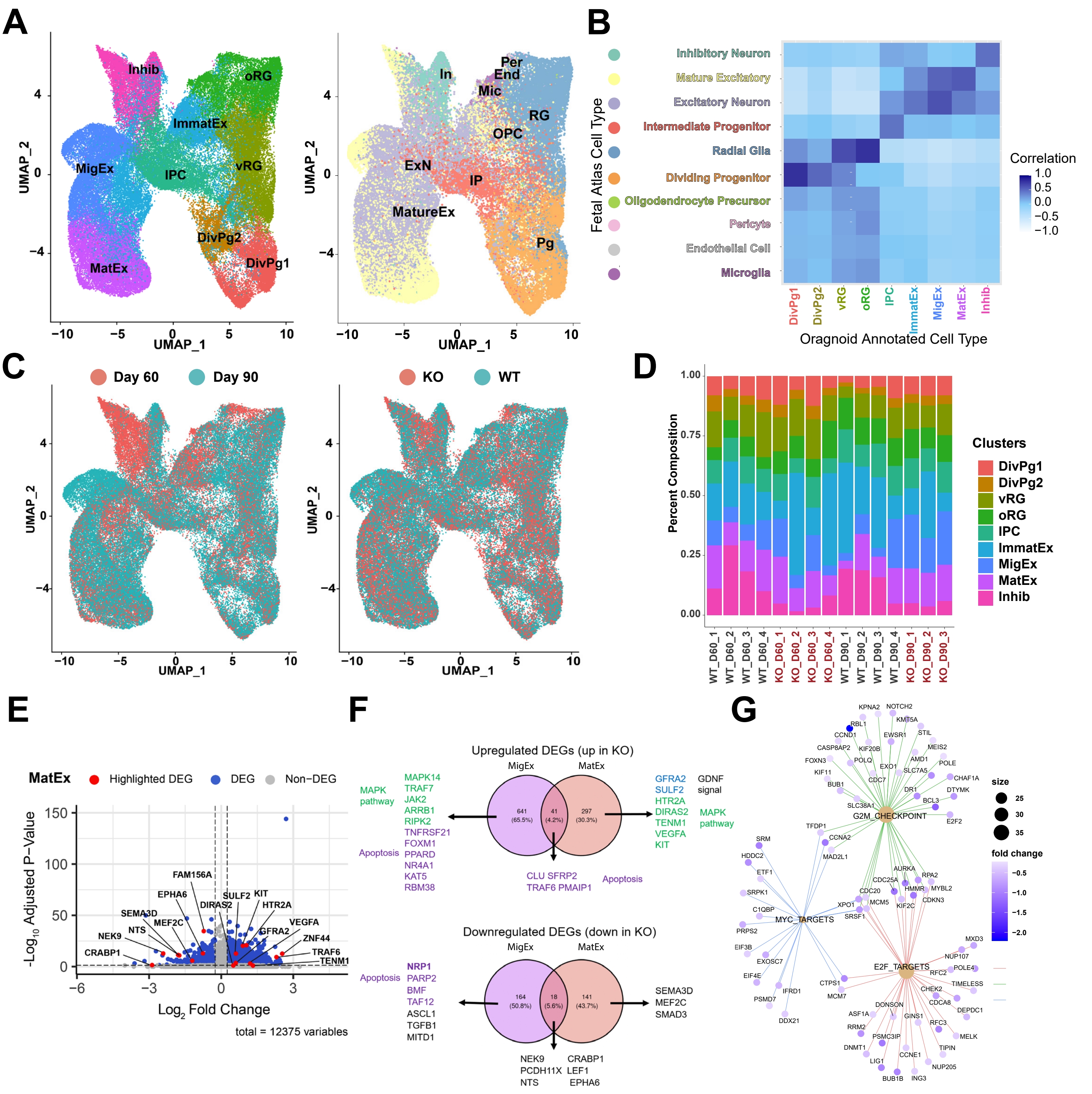

Figure S8 (Qian et al.)

SUPPLEMENTARY TABLE

Table S1. Clinical Information of Presented Subjects. Related to **Figure 1 and S1**.

SUPPLEMENTARY FIGURES

Figure S1. Mapping and Sequencing of *KIF26A* Pathogenic Variants; Related to Figure 1.

(A-E) Confirmation and segregation analysis of *KIF26A* variants in unaffected and affected individuals of enrolled families using Sanger sequencing.

(F) Sequence alignment of the amino acids surrounding the mutations from human *KIF26A* and orthologs across species, showing high degree of conservation.

(G) Predicted helices and strands of KIF26A protein architecture. Unannotated regions display conserved and transiently ordered islands, See **STAR Methods**. The locations of compound heterozygous mutations in the human KIF26A sequence are noted by asterisks above the domain graphic, and with the exception of R721C that is located in the motor domain, all fall in the unstructured chain between D2 and D3.

(H) Predicted structures of globular domains in KIF26A protein. Domain D1 is a compact 3-helix bundle with a cluster of 4 Cys residues at one end that likely coordinate Zn2+, that structurally resembles the rare IMA1 module that has been seen in nuclear envelope membrane proteins (like yeast IMA1 or human TMEM210). Domain D2 is the centrally-located and microtubule-binding motor domain that closely matches other kinesin structures (here, for comparison is PDB 4BN2, for KIF15, with bound Mg2+ and ADP). The C-terminal domain D3 has a helix-loop-helix fold that maps to the region of KIF26A that interacts with FAK.

Figure S2. Functional Characterization of KIF26A Patient Variants; Related to Figure 1.

(A) HEK293T cells transfected with WT and variant *KIF26As* with a mCherry fluorescent tag, co-immunostained for KIF26A and microtubule marker β3 Tubulin. KIF26A expression is only detectable in transfected mCherry$^+$ cells, consistent with the low baseline endogenous expression of KIF26A in HEK293T cells. Scale bar = 10 μm.

(B) Microtubule depolymerization assay on SHSY5H cells transfected with WT and variant KIF26A. Transfected SHSY5Y cells were treated with microtubule depolymerizer Nocodazole (10μM for 15 min) before fixation. Diffused staining patterns of Acetylated Tubulin and β3 Tubulin indicated microtubule depolymerization. Scale bar = 10 μm.

(C) Machine-learning based prediction of the impact of patient missense variants to protein stability using MUpro Predictor (Cheng et al., 2006). Prediction of the sign of energy change using Support Vector Machines (SVM) and neural networks: method used, effects of mutation on protein stability, and a confidence score between -1 and 1 to measure the confidence of the prediction. A score less than 0 means the mutation decreases the protein stability. The smaller the score, the more confident the prediction is. Conversely, a score more than 0 means the mutation increases the protein stability. The bigger the score, the more confident the prediction is.

(D) Representative images of acetylated tubulin (AceTub) and detyrosinated tubulin (DtTub) immunostaining for SHSY5Y transfected with scrambled shRNA and *KIF26A* shRNA1 and shRNA2. GFP-labeled *KIF26A* shRNA1 and 2 transfected cells have reduced fluorescent intensity than neighboring untransfected cells. Only transfected cells are analyzed in **(E).** Scale bar = 10 μm.

(E) Quantification for the fluorescent intensity of acetylated tubulin and detyrosinated tubulin in transfected cells normalized to the area of the cell body. Values represent Mean ± S.D. (n = 34 cells for scrambled, 25 cells for KIF26A shRNA1 and 2 from 10 areas of views. Student's t-test, ***, $p < 0.0005$).

Figure S3. Additional Characterization of Kif26a in Embryonic Mouse Brain. Related to Figure 3.

(A) Kif26a is expressed in the CP and IZ of E16.5 mouse cortex, overlapping with neuronal markers CTIP2 and SATB2. Insets show magnified view. Scale bar = 200μm, inset = 100μm.

(B) Validation of *Kif26a* knockdown with qPCR in transfected N2A cells. Values represent Mean ± S.D. (student's t-test: ***, $p < 0.0005$).

(C) Representative images of *In utero* electroporation (IUE) of control scrambled shRNA (left) and Kif26a shRNA (right) in mouse cortex at E13.5 and analyzed at E17.5 (E13.5-17.5). Insets of magnified view show electroporated cells are SATB2^{+} neurons. Scale bar = 100μm (top), = 50μm (bottom).

(D) IUE of control plasmid (scrambled shRNA) and CMV-human *KIF26A* overexpression (OE) plasmid into mouse cortex at E13.5 and analyzed at E17.5. Insets of magnified view show electroporated cells are SATB2^{+} neurons. Scale bars = 100μm.

(A) Quantification of the laminar distributions of electroporated cells in the cortex, similar to **Figure 3C**. The cortex was evenly divided into 10 bins from basal (bin 1) to apical (bin 10) surfaces and the cell distribution was normalized by the total number of electroporated cells in the analyzed area. Values represent Mean ± S.D. (n = 7 brains for control, n = 6 for hKIF26A OE).

Figure S4. *Kif26a* KD Does Not Affect Cell Death. Related to Figure 3.

(A) Representative images showing cell death in the cortex after IUE labeled by TUNEL staining at E13.5-17.5. Scale bar = 100μm.

(B) Quantification of the dead cell density labeled by TUNEL in electroporated mouse brains at E13.5-17.5. Values represent Mean ± S.D. (n = 5 brains; Student's t-test, N.S., no significant difference).

(C) Representative images showing cell death in the cortex after IUE labeled by TUNEL staining at E13.5-15.5. Bottom show magnified. Scale bar = 100μm, top; = 50 μm bottom.

(D) Quantification of the dead cell density labeled by TUNEL in mouse brains electroporated with scrambled shRNA or *Kif26a* shRNA at E13.5-15.5, and E15.5 mouse brains without injection and electroporation. Values represent Mean ± S.D. (n = 7 brains for scrambled, 5 brains for *Kif26a* KD, 4 brains for no injection. Student's t-test, N.S., no significant difference).

Figure S5. *KIF26A* KO iPSC Line Characterization. Related to Figure 4.

(A, B) Summary table (A) and target sequence validation (B) of successful introduction of frame-shift mutation in *KIF26A* KO iPSC lines. Sanger sequence view showing edited and wild-type (control) sequences in the region around the guide sequence. The horizontal black underlined region represents the guide sequence. The horizontal red underline is the PAM site. The vertical black dotted line represents the actual cut site.

(C) KO lines have normal karyotype after edit examined by KaryoStat.

Figure S6. Characterization of *KIF26A* KO Forebrain Organoids. Related to Figures 5.

(A) Schematic summary of the protocol to generate forebrain organoids. AWEB, AggreWell™ Embryonic Body (EB) Formation Media; CHIR, CHIR99021; SB, SB431542. See **STAR Methods** for details.

(B) Representative phase contrast images for different stages of forebrain organoid differentiation. Scale bar length is indicated on the images.

(C) Representative tiled confocal images of forebrain organoid at Day 70. Dashed lines delineates the boundries seperating CP, SVZ and VZ. Scale bar = 100μm.

(D) Representative image showing KIF26A expression in neuron but not progenitors in forebrain organoid. Scale bar = 100μm.

(E) *In situ* hybridization for *KIF26A* on Day 60 WT organoid. Scale bar = 100 μm.

(F) *KIF26A* KO organoids have normal neurogenesis. Scale bars = 100μm.

(G) Representative images of PH3 immunostaining in WT and KO organoids. Scale bars = 100μm.

(H) Quantitative analysis of density of dividing $SOX2^{+}$ $TBR2^{-}$ vRG (top) and $TBR2^{+}$ IPC (bottom) visualized by PH3 immunostaining in the of WT and KO organoids. Values represent Mean ± S.D. (n = 10 organoids. Student's t-test, N.S., no significant difference).

(I) Quantitative analysis of vRG cleaveage angle in the VZ of WT and KO organoids. Cleavage angle is measured as the angle of dividing cell division axis against the apical surface of the VZ. Values represent Mean ± S.D. (Same samples as H).

Figure S7. Additional Characterization of Radial Migration and FAK Inhibition in Organoids. Related to Figures 5 and 6.

(A, B) Representative images showing EdU-labeled neuron neurons express KIF26A in WT organoid **(A)** but not in KO organoid **(B)**. Inset show magnified view of the CP area. Inset width = 50μm. Scale bars = 100μm.

(C) Representative images showing the laminar distribution of EdU labeled cells in Day 70+8 WT and KO forebrain organoids. Scale bars = 50μm.

(D) Phosphorylated FAK (pFAK Tyr576/577) is higher in the CP of KO organoids than WT organoids. Scale bar = 20μm.

(E) Representative images showing apoptosis in Day 60 WT (left) and KO (right) organoids treated with DMSO or 0.5 μM GSK2256098 (GSK) for 8 days. Dashed lines delineate the borders between the CP, SVZ and VZ. Scale bar = 100μm.

(F) Quantification of the density of apoptotic cells in the VZ, SVZ and CP layers of WT and KO organoids with GSK or DMSO treatement. Values represent Mean ± S.D. (n = 8 organoids from two pairs of isogenic lines. Student's t-test, ***, $p < 0.0005$; N.S., no significant difference).

Figure S8. Additional Bioinformatic Analyses of Brain Organoid ScRNAseq. Related to Figure 7.

(A) Graph-based clustering of single cells from WT and KO brain organoids (left) with cell type identity transferred (right) from human fetal cortex atlas (Fan et al., 2018; Nowakowski et al., 2016; Polioudakis et al., 2019; Zhong et al., 2018) based on transcriptome similarities.

(B) Correlation matrix comparing clusters annotated in brain organoids and cell types in human fetal cortex atlas.

(C) Single cells from different libraries integrate well between ages (Day 60 and Day 90) and cell lines (WT and KO).

(D) The cell-type compositions across libraries (one organoid per library) are similar to each other, demonstrating consistency between individual organoids. *KIF26A* KO did not significantly alter the cell-type composition.

(E) Volcano plot showing differentially expressed genes (DEGs) between WT and KO cells in maturing excitatory neurons. Significant DEGs with adjusted p-value < 0.05, and Log_2 fold change > 0.25 or < -0.25, are shown in blue. Selected DEGs involved in neuronal survival and apoptosis are highlighed in red.

(F) Venn diagrams showing the overlap of significant (adjusted p-value < 0.05) DEGs in migrating and maturing excitatory neurons. Selected DEG with known involvement in neuronal surival and apoptosis are highlighted.

(G) Network plots for GSEA across migrating and maturing excitatory neurons between KO and WT. G2M_CHECKPOINT, E2F_TARGETS and MYC_TARGETS_V1 are the only three significantly changed (adjusted p-value < 0.05) pathways among the 50 Hallmark pathways. For each pathway, and the number of genes in each pathway is represented by node size. Nodes are connected by lines that represent individual genes in the data set which are common to multiple nodes.
